# Mast cell desensitization inhibits calcium flux and aberrantly remodels actin

**DOI:** 10.1172/JCI87492

**Published:** 2016-09-26

**Authors:** W.X. Gladys Ang, Alison M. Church, Mike Kulis, Hae Woong Choi, A. Wesley Burks, Soman N. Abraham

**Affiliations:** 1 Department of Molecular Genetics and Microbiology, Duke University Medical Center, Durham, North Carolina, USA.; 2 GlaxoSmithKline, Rare Diseases Unit, Research Triangle Park, North Carolina, USA.; 3 Department of Pediatrics, University of North Carolina at Chapel Hill, Chapel Hill, North Carolina, USA.; 4 Department of Pathology and; 5 Department of Immunology, Duke University Medical Center, Durham, North Carolina, USA.; 6 Program in Emerging Infectious Diseases, Duke–National University of Singapore, Singapore.

## Abstract

Rush desensitization (DS) is a widely used and effective clinical strategy for the rapid inhibition of IgE-mediated anaphylactic responses. However, the cellular targets and underlying mechanisms behind this process remain unclear. Recent studies have implicated mast cells (MCs) as the primary target cells for DS. Here, we developed a murine model of passive anaphylaxis with demonstrated MC involvement and an in vitro assay to evaluate the effect of DS on MCs. In contrast with previous reports, we determined that functional IgE remains on the cell surface of desensitized MCs following DS. Despite notable reductions in MC degranulation following DS, the high-affinity IgE receptor FcεRI was still capable of transducing signals in desensitized MCs. Additionally, we found that displacement of the actin cytoskeleton and its continued association with FcεRI impede the capacity of desensitized MCs to evoke the calcium response that is essential for MC degranulation. Together, these findings suggest that reduced degranulation responses in desensitized MCs arise from aberrant actin remodeling, providing insights that may lead to improvement of DS treatments for anaphylactic responses.

## Introduction

Anaphylaxis is a severe form of allergic reaction resulting in systemic symptoms, including respiratory, gastrointestinal, or cutaneous manifestations. This may include hypotension, hypoxia, and poor end-organ perfusion (1). This allergic adverse reaction is typically precipitated when IgE-bound mast cells (MCs) come in contact with allergens, resulting in extensive degranulation of these cells. Increasingly, these reactions are directed at antigenic moieties that are routinely encountered in foods or in therapeutic drugs. Thus, approaches to treat or avoid potentially deadly reactions to these allergens are sorely needed. Rush desensitization (DS) is a widely used clinical protocol that rapidly enables allergic individuals to tolerate various foods or drugs to which they are hyperresponsive. The procedure involves exposing reactive subjects serially to increasing doses of the relevant antigen (Ag) over a short interval of time, typically with minutes or hours between doses, which renders the individual temporarily hyporesponsive to the Ag (2). Patients allergic to crucial antibiotics (3) or chemotherapy (4) are routinely desensitized by DS protocols before they are exposed to the therapeutic agent. Oral DS has also been used to allow patients to tolerate the first dose of immunotherapy regimens for certain food allergies (5). In spite of its widespread usage, the underlying mechanism of DS remains to be resolved. This limitation has severely hampered the development of alternate and perhaps more convenient approaches to tolerize reactive individuals.

MCs are now widely believed to be the primary target of the DS protocol, as reactivity to MC-dependent skin prick tests is reduced or sometimes disappears after desensitization (6). These cells constitutively express high levels of FcεRI, the high-affinity receptor for IgE. Upon allergen (antigen)-mediated activation, MCs rapidly release a collection of inflammatory mediators that are prestored within their granules. Many of these prestored mediators, such as histamine, serine proteases, and heparin, have been implicated in the pathology of anaphylactic shock (reviewed in refs. 7, 8). Additionally, activated MCs mediate de novo synthesis and secretion of a wide range of mediators that exacerbate systemic inflammation in multiple ways, including the recruitment of other inflammatory cell types such as neutrophils (9) and eosinophils (10). These prestored and de novo–synthesized mediators of MCs collectively account for much of the pathology associated with severe allergic reactions.

MC signaling events upon allergic challenge begin with the engagement of IgE bound in the MC surface by the Ag. Aggregation of receptors by cross-linking with Ag stabilizes their association with cholesterol-rich lipid rafts, which function as signaling platforms for further downstream signaling. Within these rafts, activation of Src family kinases and phosphorylation of immunoreceptor tyrosine-based activation motifs of FcεRI (11) by LYN kinase initiate a tyrosine phosphorylation cascade and the recruitment of the signaling adaptor LAT (12). This culminates in the activation of PLCγ, which catalyzes the conversion of phosphatidylinositol-4,5-phosphate (PIP_2_) to the second messengers inositol trisphosphate (IP_3_) and diacylglycerol. Binding of IP_3_ to receptors on the endoplasmic reticulum results in the emptying of intracellular Ca^2+^ stores and subsequent store-operated Ca^2+^ entry (SOCE), which results in the sustained elevation of intracellular Ca^2+^ required for exocytosis of MC granules (13). A second, LAT-independent pathway via the kinase FYN also contributes synergistically to degranulation (14). Upon FcεRI cross-linking, MCs also undergo dramatic morphological changes orchestrated by dynamic reorganization of the cytoskeleton. The actin cytoskeleton has been implicated in regulatory exocytosis in a variety of cell types, playing often complex and contradictory roles (reviewed in ref. 15). In MCs (16) and other secretory cell types (17), cortical actin is thought to be a barrier to exocytosis, and has to be redistributed before fusion of membrane-bound granules to the plasma membrane can occur. Polymerized actin can also act as an anchor for secretory vesicles (18) as well as a track for actively trafficking secretory vesicles before exocytosis (15). Importantly, actin may play negative regulatory roles in cellular signaling, and its reorganization is required for cell activation events such as the initiation of B cell receptor signaling (reviewed in ref. 19). Additionally, actin can serve as a crucial modulator of Ca^2+^ responses in hematopoietic cells, which require SOCE for a variety of downstream effector responses. In T cells, for example, inhibiting actin polymerization sustains elevated intracellular Ca^2+^ levels, augmenting cytokine responses (20). The actin regulator WAVE2 has also been reported to be important for SOCE in T cells (21). In MCs, several studies using global actin inhibitors (22, 23) as well as a recent study using mice deficient in the actin regulatory protein drebrin 1 (24) have pointed to the pivotal role of the actin cytoskeleton in influencing Ca^2+^ responses to IgE/antigen.

Although several recent studies have attempted to examine the underlying mechanisms regarding DS of MCs, there is as yet no consensus in the literature. Some reports have attributed MC hyporesponsiveness following DS to depletion of cell surface IgE following internalization of IgE/FcεRI complexes (25), while others have reported the opposite, namely reduced FcεRI internalization and impaired early and late MC responses in desensitized MCs compared with untreated MCs (26). In view of the discordance in opinion regarding the underlying basis for DS, we initiated a study to address this question. We observed that in spite of active endocytosis of IgE/FcεRI complexes during DS, appreciable amounts of residual IgE molecules capable of binding Ag were still present on the surface of desensitized MCs, indicating that the hyporesponsiveness of these cells to Ag was not due to depletion of surface IgE. Furthermore, FcεRI in desensitized MCs was still capable of signal transduction, which was inconsistent with previous reports implying abrogation of IgE-mediated signaling following complete desensitization of MCs. Significantly, we found that the actin cytoskeleton in MCs was markedly reorganized during DS, and this alteration appeared to negatively impact Ca^2+^ mobilization, a key requirement for MC degranulation, following FcεRI-mediated signaling. This observation implicates the displacement of the cellular actin cytoskeleton as the underlying reason for MC desensitization following DS.

## Results

### Establishing in vivo and in vitro models of DS.

**Figure 1:**
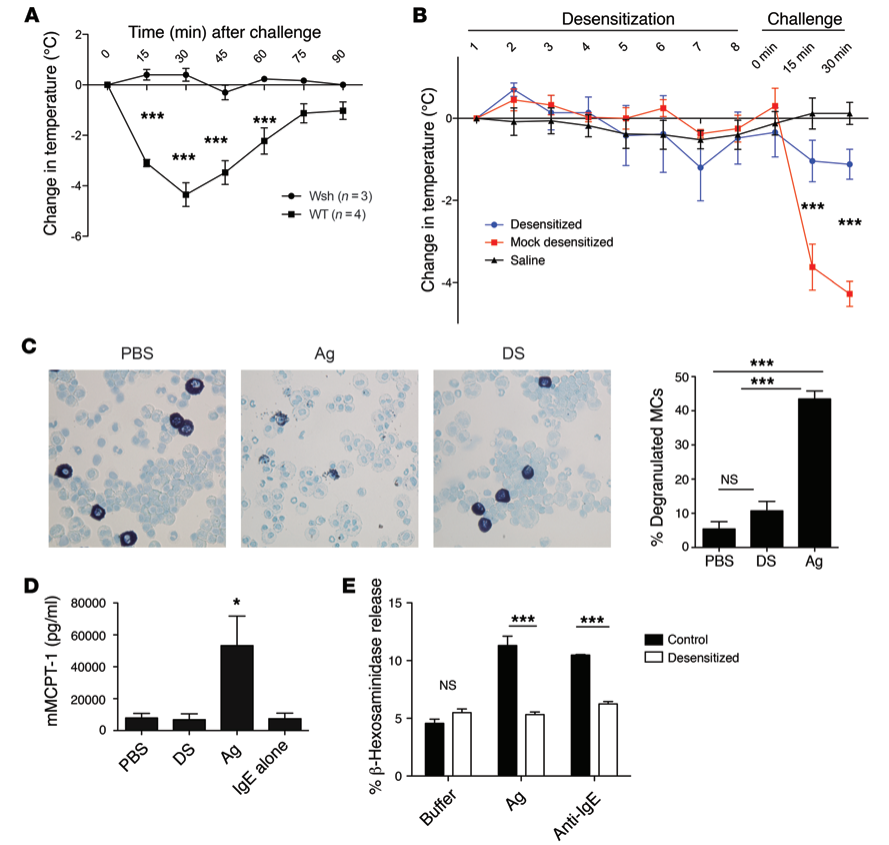
In vivo and in vitro models of desensitization. (**A**–**D**) Mice were sensitized i.p. with 10 μg IgE and challenged i.p. with 500 μg TNP-OVA. Temperatures were measured rectally and expressed as change in initial temperature. (**A**) MC-deficient Wsh mice and WT mice were challenged with Ag and monitored for 90 minutes (*n* = 3–4). (**B**) Mice were orally desensitized or mock treated for 8 doses, each spaced 30 minutes apart. Thirty minutes after the last dose, mice were challenged with Ag and rectal temperatures recorded every 15 minutes. Mice were given saline throughout (*n* = 5). (**C**) Peritoneal lavage was obtained at the end of desensitization or equivalent period of i.p. Ag challenge or PBS treatment (*n* = 4–5 per group). Lavage was cytospun and stained with toluidine blue, and the percentage of degranulated MCs was counted in 5 random fields per cytospin. Representative images at ×40 magnification are shown. (**D**) Mice were bled at the end of desensitization or Ag challenge as in **B** and serum analyzed via ELISA for MCPT-1 (*n* = 5). 5 × 10^5^ IgE-sensitized BMMCs in triplicate were desensitized or control treated, then challenged with buffer, 10 ng/ml Ag, or 0.5 μg/ml anti-IgE. β-Hexosaminidase release was measured and expressed as a percentage of total. Data were analyzed via repeated-measures ANOVA (**A** and **B**), 1-way ANOVA (**C** and **D**), or 2-way ANOVA (**E**). Error bars represent SEM. *P < 0.05, ***P < 0.001.

To address the underlying basis for MC desensitization, we first sought to establish representative in vivo and in vitro models of DS. Before establishing an in vivo DS protocol, it was necessary to develop and validate a model of passive anaphylaxis with demonstrable MC involvement. Briefly, WT and MC-deficient mice (Kit^W-sh^/Kit^W-sh^ mice, hereafter referred to as Wsh mice) were sensitized with 10 μg anti-TNP (trinitrophenyl) IgE i.p., then challenged i.p. with 500 μg TNP-OVA. Rectal temperatures were measured as an indication of anaphylaxis. MC-deficient Wsh mice were refractory to anaphylaxis, while WT mice showed a drastic drop in temperature 15–30 minutes after challenge (Figure 1A). Using this model, we sought to establish an oral DS protocol to protect against anaphylaxis. We gavaged IgE-sensitized WT mice with increasing doses of Ag (Table 1) or with equivalent volumes of PBS at 30-minute intervals. During the desensitization process, all groups showed no significant changes in core body temperature. At the end of the desensitization or control treatment, mice were challenged i.p. with 500 μg of TNP-OVA in PBS. Control (PBS-treated, IgE-sensitized) mice showed a sharp decrease in core body temperature after challenge, indicating anaphylaxis, while desensitized mice subjected to DS exhibited very limited changes in temperature in response to challenge, which were not significantly different from those in unsensitized mice (Figure 1B). To confirm that there was limited MC degranulation during the desensitization process, we stained peritoneal lavages obtained from control, Ag-challenged, or desensitized mice with toluidine blue, which stains MC granules a deep purple color. As expected, Ag-challenged mice had a significantly higher percentage of partially or fully degranulated MCs as observed via microscopy, while IgE-alone and desensitized mice had mostly fully granulated MCs (Figure 1C). To further confirm MC involvement in our model, mice were bled 30 minutes after Ag challenge of desensitized or control mice for serum analysis of murine mast cell protease 1 (mMCPT-1), a known MC granule–associated chymase released during anaphylaxis (27). Mice that had been sensitized with IgE but were not Ag challenged (IgE alone), as well as unsensitized PBS-injected mice (PBS), had very low levels of serum mMCPT-1 (Figure 1D). As expected, Ag-challenged mice had very high levels of serum mMCPT-1. Importantly, desensitized mice had levels of serum mMCPT-1 not significantly different from those seen with baseline PBS despite oral administration of Ag during desensitization and subsequent i.p. challenge. Thus, our in vivo model of DS was effective, as it protected mice from anaphylaxis by preventing MC degranulation.

Next we sought to develop an in vitro model for DS that was applicable for murine bone marrow–derived mast cells (BMMCs) as well the RBL-2H3 cell line (Figure 1E and Table 2). The model we developed was adapted from Sancho-Serra et al. (26). MC degranulation was assessed by measurement of extracellular levels of the granule component β-hexosaminidase. The extent of hexosaminidase release (degranulation) was expressed as a percentage release over total hexosaminidase present in cells. IgE-sensitized BMMCs were either desensitized or control treated before cross-linking of IgE/FcεRI with anti-IgE or Ag. Upon Ag or anti-IgE challenge, control MCs degranulated significantly (Figure 1E), while desensitized MCs exhibited limited degranulation response to activation by either Ag or anti-IgE. Having validated the efficacy of this in vitro model of DS, we used this model for our subsequent studies to elucidate the underling basis for MC desensitization.

### Functional IgE remains on the surfaces of desensitized MCs.

**Figure 2:**
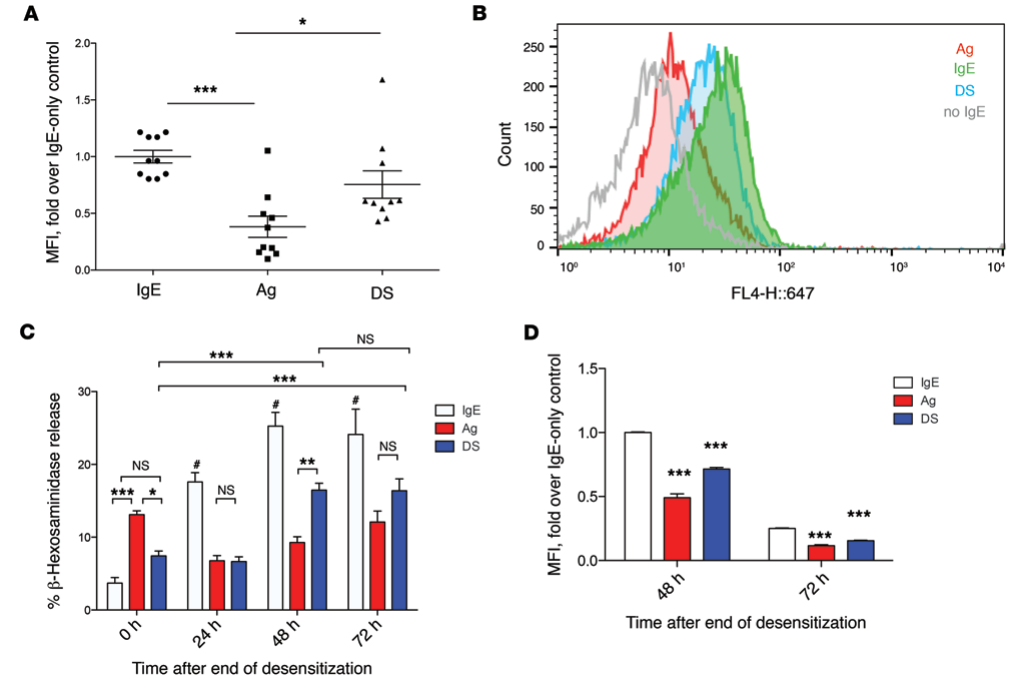
Complete depletion of surface IgE does not occur with desensitization. (**A**) IgE-sensitized mice were desensitized (DS), challenged with antigen (Ag), or injected with PBS (IgE). At the end of desensitization, mice were sacrificed for peritoneal lavage. Lavage cells were incubated with A647-labeled TNP-OVA for 1 hour on ice, then washed 5 times with PBS before fixation and assay via FACS. Data were expressed as fold median fluorescence intensity (MFI) over IgE alone. Data are combined from 2 experiments (*n* = 10). (**B**) 5 × 10^5^ IgE-sensitized BMMCs per sample in quintuplicate were desensitized (blue), Ag challenged (red), or untreated (green), then labeled with A647–TNP-OVA as in **A**. BMMCs without IgE were also incubated with labeled TNP-OVA as control (gray). A histogram representative of at least 3 experiments is shown. (**C**) 5 × 10^5^ BMMCs per sample of IgE-alone (IgE), Ag-challenged (Ag), or desensitized (DS) cells were washed 3 times in PBS before resuspension in 500 μl RPMI without IgE. Zero to 72 hours later, cells were collected and challenged with 10 ng/ml Ag. Degranulation was measured using a β-hexosaminidase assay. (**D**) Control (IgE), Ag-challenged (Ag), or desensitized (DS) cells were incubated for 0 or 48 hours before being assayed for surface staining of IgE via FACS. Data are representative of at least 3 independent experiments. Data were analyzed via 1-way (**A**) or 2-way (**C** and **D**) ANOVA. Error bars indicate SEM. *P < 0.05, **P < 0.01, ***P < 0.001, ^#^*P* < 0.001 compared with all other groups.

Previous studies have suggested that MC desensitization was attributable to the internalization of IgE antibodies during this process. If this were the case, then there should be limited IgE left on these cell surfaces capable of binding sufficient Ag for activation. To test the residual Ag-binding ability of desensitized cells, we collected peritoneal lavages from IgE-sensitized mice that had been desensitized (DS), Ag challenged (Ag), or unchallenged (IgE). Peritoneal cells obtained from the lavage were incubated with Alexa Fluor 647–labeled (A647-labeled) TNP-OVA ex vivo (Figure 2A), and the extent of Ag binding was assessed by FACS, with gating for c-kit^+^ cells, which in the peritoneal cavity are all MCs (28). As expected, c-kit^+^ cells from IgE-sensitized mice bound high amounts of fluorescent Ag, due to high levels of surface IgE. MCs from Ag-challenged mice had the lowest fluorescence, reflecting internalization of IgE resulting from Ag challenge, a well-described consequence of IgE-mediated MC activation (29). Surprisingly, c-kit^+^ cells from desensitized mice bound high levels of fluorescent Ag, significantly more than Ag-challenged mice. This comparatively high binding capacity to additional Ag suggested that surface IgE was still plentiful, in spite of IgE internalized during the DS protocol. To support our findings, we repeated this experiment in vitro using BMMCs. IgE-sensitized BMMCs were desensitized, challenged with Ag, or left unchallenged, before being collected and incubated with A647-labeled TNP-OVA. After extensive washing, cells were processed for FACS (Figure 2B). As expected, based on A647 fluorescence, untreated (IgE alone) BMMCs had the highest Ag binding, as a result of having the highest surface IgE. Ag-challenged MCs showed the least Ag binding. Desensitized BMMCs showed almost as much Ag binding as IgE-only cells, though the decrease was still statistically significant. Importantly, desensitized MCs could bind significantly more Ag than Ag-challenged MCs. BMMCs without IgE, included as a negative staining control, did not bind Ag significantly. These data thus suggested that surface IgE was not completely internalized following the desensitization process, and enough IgE remained on the cell surface to bind Ag that could potentially cause degranulation. Indeed, we have found that degranulation does not decrease proportionately with decreasing concentrations of sensitizing IgE, indicating that even low amounts of surface IgE are sufficient to trigger degranulation (Supplemental Figure 1; supplemental material available online with this article; doi:10.1172/JCI87492DS1).

The residual Ag-binding capacity of desensitized MCs made us speculate that desensitized MCs could perhaps recover the ability to degranulate in response to Ag challenge, much as the effects of DS in clinical settings are temporary, with subjects regaining their reactivity to the allergen after the desensitization process is discontinued (30). In this way, we could also further demonstrate that desensitization of MCs was not due to lack of cell surface IgE by showing that recovery of Ag sensitivity can occur even without addition of fresh IgE. For this experiment, IgE-sensitized BMMCs were desensitized, challenged with Ag, or left unchallenged, then washed multiple times, resuspended in BMMC culture medium, and allowed to recover without any additional IgE included in the medium. After 0, 24, 48, or 72 hours of incubation at 37°C, MCs were challenged with 10 ng/ml of Ag. As expected, cells that were not initially challenged with Ag at 0 hours (IgE group) had the highest degranulation responses when challenged at 24, 48, or 72 hours. Notably, the degranulation responses of this group increased with time, a result that might be attributable to the upregulation of granule components induced by prolonged incubation with IgE (31). By contrast, cells challenged with Ag at 0 hours were unable to significantly degranulate to further Ag challenge until 72 hours. In desensitized cells, we determined that by 48 hours after desensitization, BMMCs had regained their ability to significantly degranulate to Ag challenge, even without addition of fresh IgE (Figure 2C). To investigate whether this recovery by 48 hours in desensitized MCs was due to recycling of IgE back to the cell surface, we incubated IgE-sensitized, Ag-stimulated, or desensitized BMMCs in IgE-free media for 0 or 48 hours before assessing surface IgE levels via FACS. As expected, unstimulated (IgE only) cells had the highest surface IgE levels for both time points, while Ag-stimulated cells had the lowest surface IgE. Overall, surface IgE declined over time in all groups. Desensitized MCs had intermediate surface IgE levels between those of Ag-treated and IgE control BMMCs, but there was no increase in surface IgE after 48 hours (Figure 2D). Therefore, we deduce that recovery likely was not due to recycling of previously endocytosed IgE back to the cell surface. To further establish that the inability of desensitized cells to respond to Ag was not due to the lack of IgE, we resensitized desensitized BMMCs with 0.5 μg/ml IgE at both 0 and 24 hours after desensitization. We did not find a difference between resensitized and untreated desensitized cells, as both groups showed little degranulation in response to Ag challenge compared with controls (Supplemental Figure 2). However, the lack of degranulation may also be due to the inability of freshly added IgE to bind to the MC surface, owing to the lack of unoccupied FcεRI. Cumulatively, these observations support the notion that absence of cell surface IgE to activate MCs is not the reason for the lack of response to Ag in desensitized cells.

### Desensitized MCs transduce signals following Ag challenge despite abrogation of degranulation.

**Figure 3:**
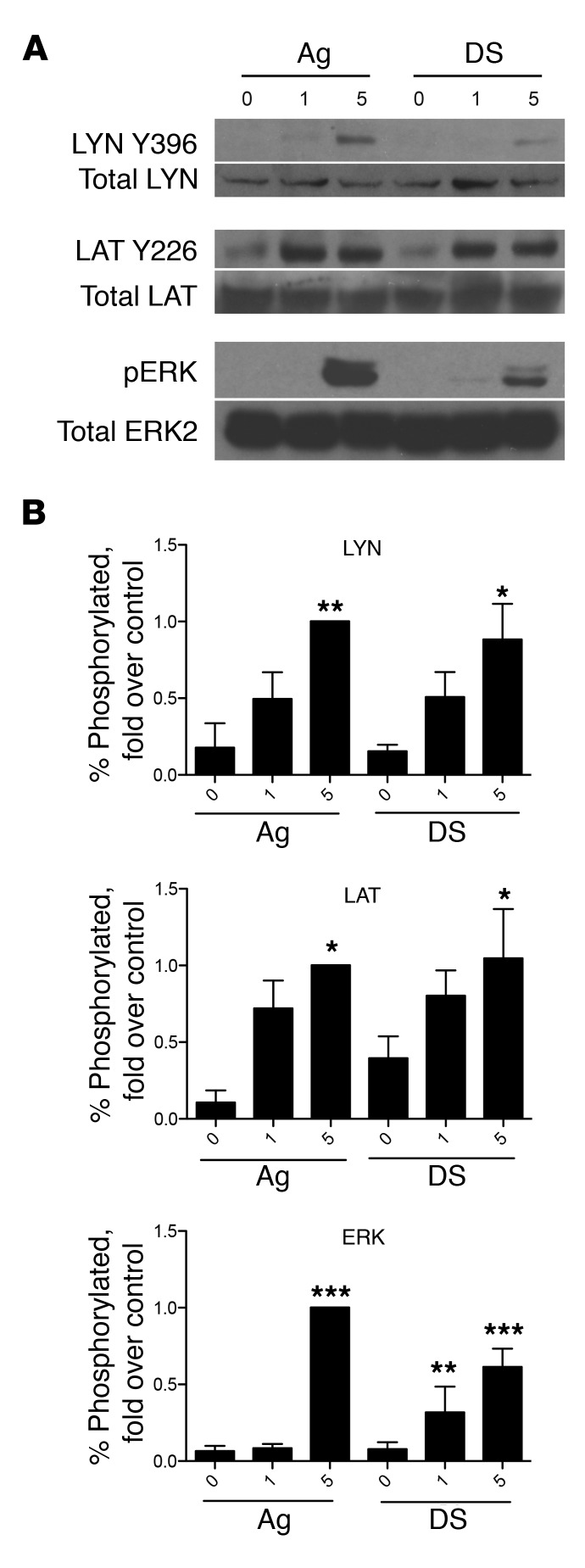
Desensitized cells have signaling responses to Ag. (**A**) 2 × 10^6^ BMMCs per sample were sensitized with IgE, desensitized (DS), or left untreated (Ag) before challenge with Ag for 0, 1, or 5 minutes. Cells were directly lysed in 4× Laemmli buffer for SDS-PAGE and immunoblotted for phospho- and total LYN, LAT, and ERK. Representative blots from 4 experiments are shown. (**B**) Densitometry was performed on blots from 4–5 experiments. Data were expressed as percentage of phosphorylated protein compared with total, then normalized to the positive control (5 minutes Ag activation). Data were analyzed via 1-way ANOVA and represent SEM. *P < 0.05, **P < 0.01, ***P < 0.001 compared with IgE group.

Since residual IgE could be detected on MC surfaces even after desensitization, we sought to identify alternate mechanisms for MC desensitization. First, we investigated whether desensitized MCs still were able to transduce signals, even though degranulation failed to occur in response to additional Ag stimulation, as shown in Figure 1E. For this, we compared the kinetics of tyrosine phosphorylation of LYN, LAT, and ERK between IgE-sensitized and desensitized BMMCs, using the activation of these proteins as indicators of signaling at different stages of the IgE/FcεRI signaling pathway. To do this, we used immunoblotting to assay the phosphorylation of these proteins at sites associated with their activation. Compared with untreated controls, Ag-stimulated MCs showed strong phosphorylation of LYN, LAT, and ERK within 5 minutes of exposure. Surprisingly, a similar pattern of phosphorylation was observed with desensitized cells challenged with Ag, with a significant increase in phosphorylated ERK, LAT, and LYN in comparison with IgE controls that was not significantly different from the increase seen in the respective Ag challenge groups (Figure 3). Thus, the inability of desensitized MCs to degranulate is not due to complete abrogation of IgE/FcεRI signaling, as key components in this pathway were activated following Ag challenge.

### Failure of desensitized MCs to degranulate in response to Ag challenge is due to their inability to mobilize Ca^2+^.

**Figure 4:**
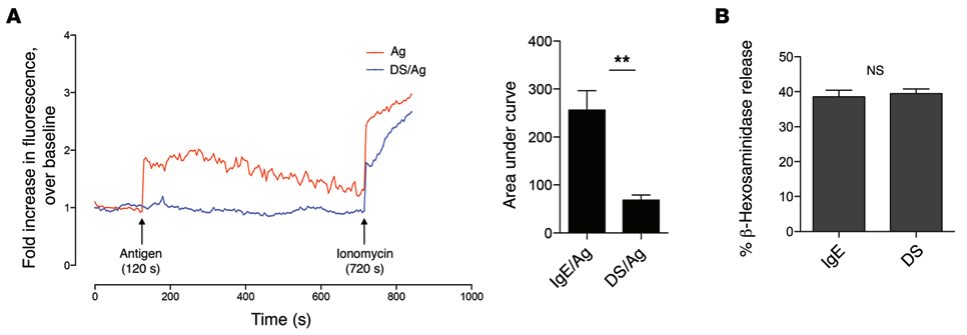
Calcium mobilization is inhibited in desensitized cells. (**A**) IgE-sensitized BMMCs were left untreated (blue) or desensitized (red). After labeling with Fluo-4, cells were challenged with Ag (arrow) for 5 minutes. Responsiveness was confirmed by addition of ionomycin. Fluorescence was normalized to baseline. A representative graph is shown. AUC analyses were averaged from at least 4 independent experiments. (**B**) 5 × 10^5^ BMMCs per sample in triplicate were desensitized or control treated before addition of 1 μg/ml ionomycin. Degranulation was measured via β-hexosaminidase assay. Data were analyzed via 2-tailed Student’s *t* test. Error bars represent SEM. **P < 0.01.

Our studies of desensitized MCs so far revealed that the block in degranulation responses must occur further downstream of these signaling substrates described above. A major requirement for MC degranulation following the initial tyrosine phosphorylation cascade is Ca^2+^ mobilization (32). Therefore, we compared Ca^2+^ responses of desensitized and control MCs following Ag challenge. For these experiments, desensitized and control MCs were loaded with the Ca^2+^-sensitive dye Fluo-4 AM, and then exposed to Ag. Changes in Fluo-4 fluorescence corresponding to changes in intracellular Ca^2+^ were assessed using a confocal microscope. In contrast to control MCs, which showed a clear, sustained Ca^2+^ response, desensitized MCs failed to exhibit any change in intracellular Ca^2+^ levels upon Ag challenge (Figure 4A). Thus we hypothesized that the inability of desensitized MCs to degranulate was attributable to their failure to mobilize Ca^2+^. To underscore this conclusion, we exposed both control and desensitized MCs to ionomycin, a Ca^2+^ ionophore that complexes with and directly transports Ca^2+^ across the cell membrane, artificially increasing intracellular Ca^2+^ levels. Ionomycin elicited comparable degranulation responses in desensitized and control MCs. Thus, artificially elevating intracellular Ca^2+^ in MCs was sufficient to overcome the block to degranulation caused by desensitization (Figure 4B).

### IgE/FcεRI–mediated signaling occurs during DS of MCs.

**Figure 5:**
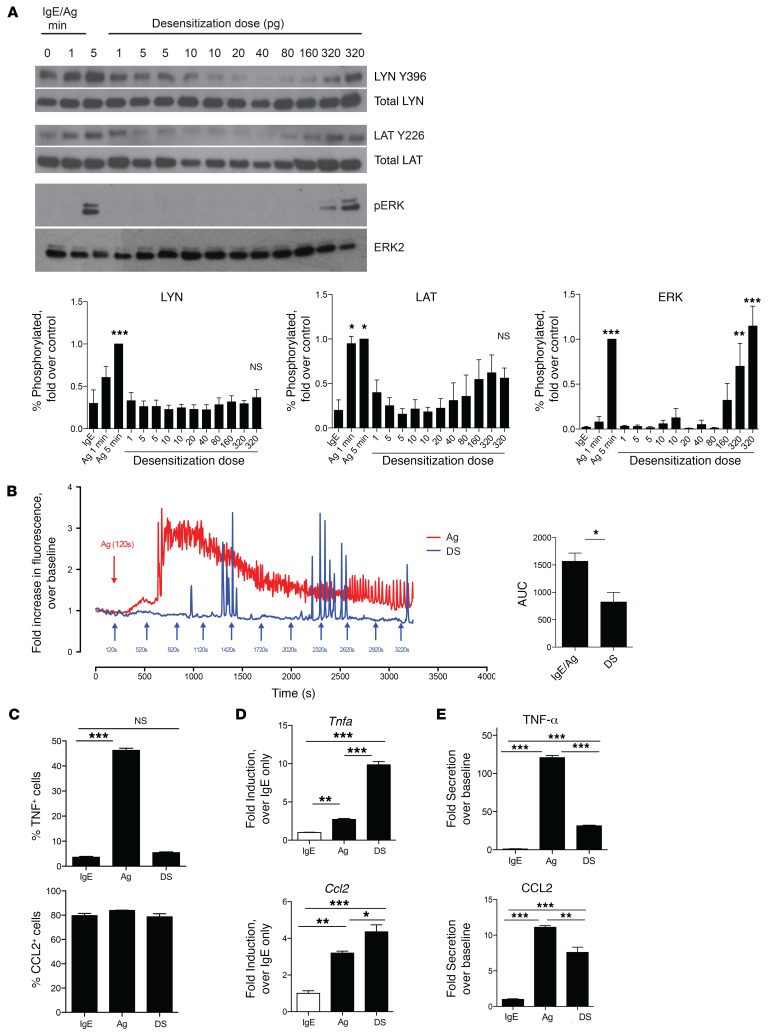
Signal transduction but no calcium mobilization during RDS. (**A**) 2 × 10^6^ BMMCs per sample were sensitized with IgE before Ag challenge for 0, 1, or 5 minutes (first 3 lanes) or desensitized (lanes 4–14), with samples collected 1 minute after each dose was added. Samples were immunoblotted for phospho- and total LYN, LAT, and ERK. Blots are representative of at least 3 experiments. For densitometry, data were expressed as percentage of phosphorylated protein compared with total, then normalized to the positive control (5 minutes Ag). Data were analyzed via 1-way ANOVA with comparisons with IgE (Dunnett’s post-test). (**B**) BMMCs were adhered to Cell-Tak–coated (Corning) glass-bottomed plates for calcium imaging. Cells were stimulated with Ag or desensitized (DS), with successive doses added every 5 minutes (arrows). AUC analyses were averaged from 4–5 independent experiments. (**C**–**E**) 5 × 10^5^ IgE-sensitized BMMCs per sample in triplicate were desensitized (DS), antigen challenged (Ag), or untreated (IgE). (**C**) BMMCs were incubated with brefeldin A for 8 hours before intracellular staining for FACS. (**D**) RNA was isolated from cell pellets for cDNA preparation. Quantitative RT-PCR was performed using primers for *Ccl2* and *Tnfa* and normalized to *Actb* expression. (**E**) Cell supernatants after 24 hours were analyzed for CCL2 and TNF-α secretion. Data were expressed as fold over IgE-only controls. Data were analyzed via 1-way ANOVA (**A** and **C**–**E**) or Student’s *t* test (**B**). Data represent SEM. *P < 0.05, **P < 0.01, ***P < 0.001.

Since we observed no degranulation during the DS protocol, we predicted that escalating Ag doses could elicit signal transduction without Ca^2+^ mobilization. To test this notion, we prepared cell lysates sequentially from MCs 1 minute after each exposure to an escalating dose of Ag and subjected them to SDS-PAGE and Western blotting. As in Figure 3, we immunoblotted MC lysates for LYN, LAT, and ERK phosphorylation. Control MCs activated for 1 or 5 minutes were included for comparison (Figure 5A). As desensitization progressed, we observed increased phosphorylation of LAT and ERK, but not LYN, which was proportional to the cumulative Ag dose, though this change was statistically significant only for ERK when densitometry was performed (Figure 5A). Next, we investigated Ca^2+^ mobilization during desensitization. Compared with MCs stimulated with Ag for the same length of time, desensitized cells exhibited limited Ca^2+^ signals, with observable spikes in fluorescence, but no indication of sustained Ca^2+^ mobilization (Figure 5B).

Since ERK, which is a key regulator of cytokine expression in MCs (33), was highly phosphorylated during desensitization, we investigated whether any de novo synthesis of select cytokines occurred during the protocol. To address this question, unstimulated, Ag-stimulated, or desensitized MCs were stained for intracellular CCL2 and TNF-α for FACS analysis. CCL2 seemed to be mostly preformed in all cells, but we found that there was significant de novo synthesis of TNF-α in Ag-stimulated cells following Ag exposure, as evidenced by the increase in TNF-α–positive cells from baseline levels. In contrast, there was no difference in desensitized cells (Figure 5C). Unexpectedly, when we analyzed mRNA transcript levels of *Tnfa* and *Ccl2*, desensitized cells had high levels of transcripts for both, with an increase from baseline that was even significantly greater than that of Ag-stimulated MCs (Figure 5D). To confirm whether secretion of cytokines was occurring, we assayed culture supernatants 24 hours after control treatment, desensitization, or Ag stimulation to analyze the cumulative cytokine release via ELISA (Figure 5E). As expected, Ag-stimulated cells showed high secretion of both TNF-α (>100-fold over baseline) and CCL2 (>10-fold over baseline). Interestingly, desensitized cells showed significantly reduced cytokine secretion compared with Ag-stimulated cells, but still showed modest production of cytokines compared with that seen with IgE alone (~30-fold over baseline for TNF-α and ~8-fold over baseline for CCL2). It thus appeared that while signaling leading to transcription of cytokines is functional, and even enhanced, during desensitization, the lack of a Ca^2+^ response might be a factor contributing to the lack of maximal protein production, as sustained Ca^2+^ is required for optimal cytokine production in other cell types (34).

### Desensitized MCs show distinct changes in F-actin distribution.

**Figure 6:**
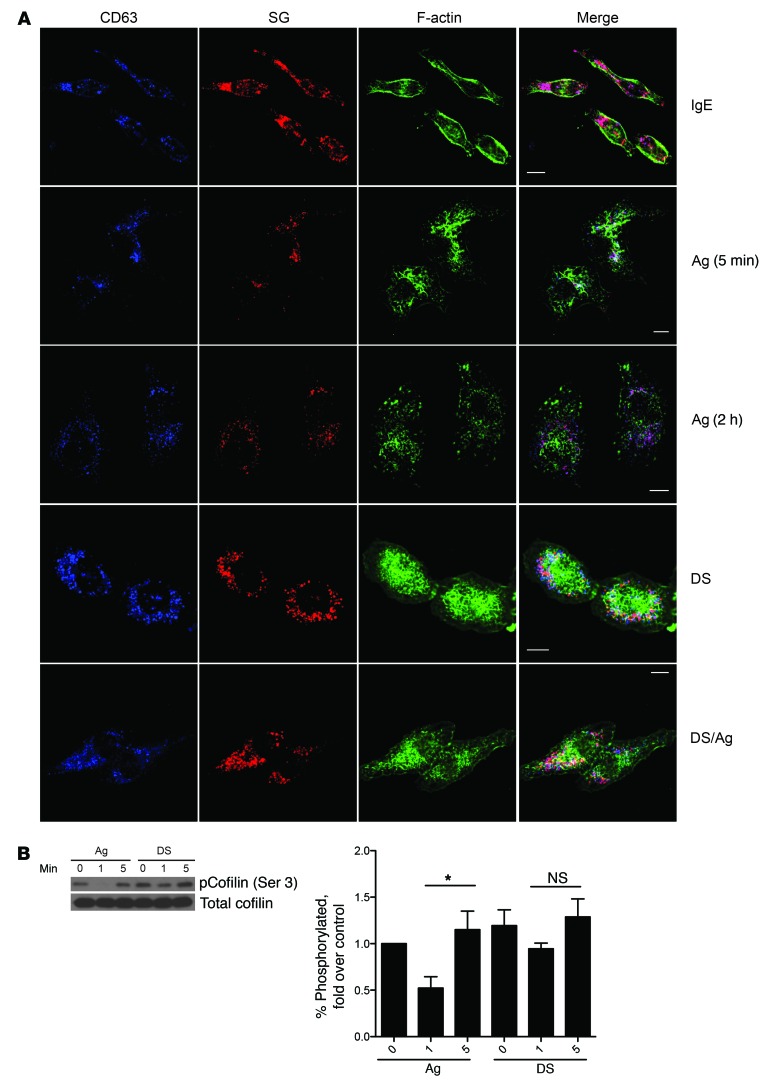
Distinct actin reorganization in desensitized cells. (**A**) Serglycin (SG)–mCherry (red) RBL-2H3 cells were plated on coverslips before desensitization and/or challenge with Ag for 5 minutes followed by fixation and staining for CD63 (blue) and F-actin (green). Images are representative of at least 3 independent experiments. Scale bars: 10 μm. (**B**) 2 × 10^6^ BMMCs per sample were desensitized (DS) or untreated (Ag) before challenge with Ag for 0, 1, or 5 minutes. Lysates were immunoblotted for phosphorylated and total cofilin. Blots represent at least 4 experiments. Densitometry data were expressed as percentage phosphorylated protein compared with total, then normalized to control (IgE). Data were analyzed via 1-way ANOVA. Data represent SEM. *P < 0.05.

If the absence of a sustained Ca^2+^ response explains the lack of MC degranulation during DS when subactivating doses of Ag were used, then why do desensitized MCs not degranulate in response to an optimal challenge dose of Ag? One possible explanation is that the desensitization process results in the redistribution of actin, an important regulator of the Ca^2+^ response. The spatial distribution of actin has been shown to be a critical determinant of Ca^2+^ mobilization in T cells (35), B cells (36), and MCs (23). To test this notion, we sought to investigate whether significant reorganization of F-actin occurred during the desensitization protocol, and consequently, whether this activity was responsible for impeding subsequent Ca^2+^ mobilization following exposure to Ag. To test this possibility, we investigated changes in F-actin distribution in IgE-sensitized MCs before and after desensitization by confocal microscopy. For these imaging studies, we substituted the MC-like cell line RBL-2H3 for BMMCs, as these cells were easy to transfect and were adherent, making them easier to image. RBL-2H3 cells stably expressing mCherry-tagged serglycin, a proteoglycan component of granules, were used to visualize granules. Unstimulated, IgE-sensitized cells had clear spindle morphology with a peripheral distribution of F-actin and strong staining for granules, as indicated by serglycin-mCherry surrounded by CD63^+^ secretory granule membranes. In Ag-stimulated cells, F-actin rapidly reorganized into large, distinct membrane ruffles by 5 minutes after challenge. Subsequently, F-actin depolymerized, and by 2 hours redistributed into small aggregates dispersed throughout the cell (Figure 6A). These cells also exhibited a flattened morphology with few intracellular granules, consistent with extensive degranulation (Figure 6A). Although desensitized MCs exhibited a flattened cell morphology, they remained fully granulated and, remarkably, exhibited extensive aggregates of F-actin throughout the cell (Figure 6A). When we examined MCs at various stages of desensitization, we found that the percentage of cells with extensive F-actin reorganization increased as the desensitization procedure progressed (Supplemental Figure 3). Thus, compared with Ag-stimulated MCs, which exhibited extensive F-actin reorganization after activation and which subsequently mostly disassembled by 2 hours, the F-actin structures in desensitized cells remained stable for at least 2 hours (Figure 6A). Notably, stimulation of desensitized MCs with normally activating doses of Ag failed to elicit appreciable changes to cellular F-actin distribution (Figure 6A), indicating an inability to evoke further mobilization of actin.

Next, we investigated the underlying basis for the stability of reorganized actin in desensitized MCs and their intractability to further remodeling in response to activating doses of Ag by focusing on the activation of distinct proteins involved in actin reorganization. Specifically, we assayed for the phosphorylation of cofilin, an actin-severing protein. Cofilin generates free actin filament ends to facilitate reorganization, and is inactivated by phosphorylation at the serine 3 position. In control MCs stimulated with Ag, cofilin was rapidly dephosphorylated 1 minute after activation and highly rephosphorylated by 5 minutes after Ag activation, suggesting a brief activation window followed by negative feedback regulation during IgE/Ag activation. However, in desensitized MCs, the dephosphorylation and rephosphorylation of cofilin in response to Ag challenge, while observable, were not significant (Figure 6B), suggesting there may be some inhibition of cofilin-mediated actin turnover, possibly explaining the stability of F-actin filaments in desensitized MCs. Thus, desensitization induces stable actin rearrangements in desensitized MCs that are unresponsive to further actin reorganization.

### Specific recruitment of actin to desensitized receptors accounts for the Ag specificity of desensitization.

**Figure 7:**
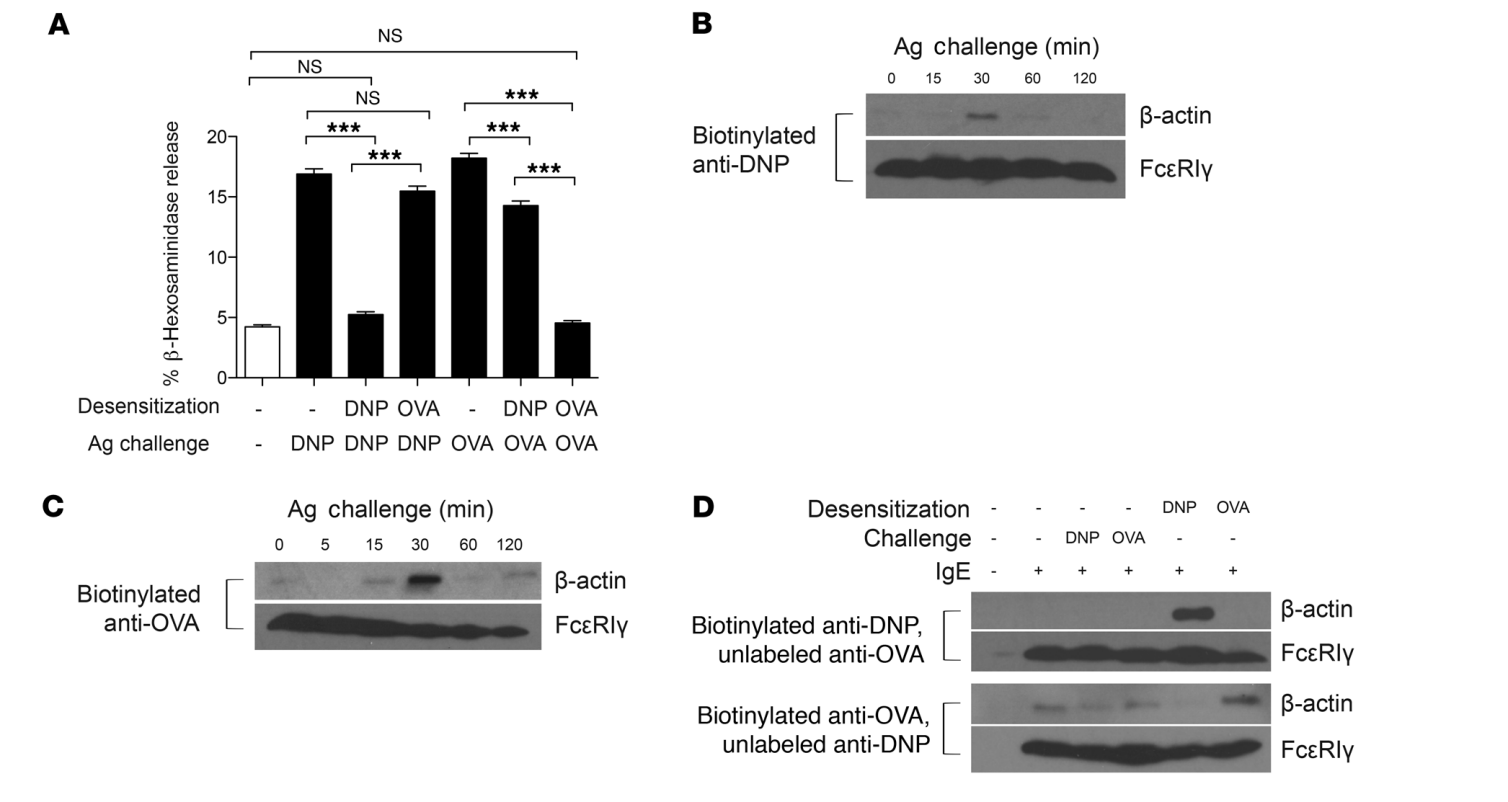
Antigen specificity of desensitization. (**A**) RBL-2H3 cells were sensitized with a 1:5 ratio of anti-DNP to anti-OVA IgE, then desensitized to DNP-HSA or OVA before challenge with DNP-HSA (10 ng/ml) or OVA (1 μg/ml). Data were analyzed via 1-way ANOVA and are representative of at least 3 independent experiments. (**B** and **C**) 10^7^ BMMCs per sample were sensitized with biotinylated IgE, then challenged with Ag for the indicated times. Lysates were immunoprecipitated with streptavidin beads and blotted for β-actin and FcεRIγ. (**D**) 10^7^ BMMCs per sample were sensitized with a mixture of biotinylated and unbiotinylated IgE, then desensitized or challenged with Ag for the same amount of time (~2 hours). Lysates were then immunoprecipitated with streptavidin beads and blotted for β-actin and FcεRIγ. Data represent SEM. ***P < 0.001.

If displacement of the cellular actin cytoskeleton is the underlying basis for MC desensitization, how does this proposed mechanism explain the characteristic Ag specificity of the desensitization protocol? To explain the antigenic specificity of the desensitization protocol, we hypothesized that the actin reorganization that occurs during desensitization must be specific to the desensitized receptor, and not to heterologous receptors bound to IgE specific for a different Ag. These heterologous complexes should then be unimpeded to trigger MC degranulation. To validate this notion, we sensitized RBL-2H3 cells with a mixture of IgE directed at 2 distinct antigens: OVA and dinitrophenyl (DNP). To demonstrate that MC desensitization was Ag specific, we desensitized MCs with one Ag and examined the degranulation response to heterologous Ag. Groups of MCs were simultaneously sensitized with anti-DNP and anti-OVA IgE antibodies, then desensitized with OVA or DNP-HSA, or left undesensitized (Figure 7A). To achieve similar degranulation levels to Ag, a 1:5 mixture of anti-DNP/anti-OVA IgE was used to sensitize cells (Supplemental Figure 4). The variously treated groups were then subjected to Ag challenge with either OVA or DNP-HSA. We found that desensitization of MCs with OVA did not significantly decrease the MC degranulation response to the heterologous Ag DNP (Figure 7A). For MCs desensitized with DNP, while there was a significant reduction in response to OVA, more than 75% of the degranulation response to OVA was still preserved. This confirms that desensitization is largely Ag specific. Previous reports have noted that cross-linking of IgE/FcεRI results in their association with the actin cytoskeleton (37–39), which might impact downstream function. We thus wondered whether a similar phenomenon could explain the Ag specificity of DS. We hypothesized that actin association with IgE/FcεRI also occurred during DS and, importantly, was specific to the desensitized receptor. Firstly, we confirmed that there was an association between actin and IgE/FcεRI following MC activation. We sensitized BMMCs with biotinylated anti-OVA or anti-DNP IgE, then challenged them with the corresponding Ag. We previously confirmed that biotinylated IgE was functional and could elicit degranulation (Supplemental Figure 5). We prepared cell lysates at various time points after Ag stimulation and immunoprecipitated the lysates using streptavidin agarose beads to pull down the biotinylated IgE and associated FcεRI and other proteins in the complex, such as β-actin. Immunoblotting revealed that β-actin was only weakly associated with IgE/FcεRI at 0 minutes, then became maximal at 30 minutes after activation, with levels of immunoprecipitated β-actin returning to steady-state levels by 2 hours (Figure 7, B and C). This finding is in agreement with earlier studies showing maximal association of the actin cytoskeleton with IgE/FcεRI approximately 30 minutes after Ag stimulation of MCs (38, 39).

Next, we investigated whether a similar association could be observed between β-actin and desensitized FcεRI following desensitization of MCs with each of the 2 antigens. For this, one of the IgE clones in the sensitization mixture (anti-OVA or anti-DNP IgE) was biotinylated, while the other was left IgE unlabeled. Thus, MCs were sensitized with a mixture of IgE where only IgE of one specificity was biotinylated but not the other. After desensitization or Ag challenge with either Ag (~2 hours of incubation), cell lysates were prepared and immunoprecipitated with streptavidin beads, which only pulled down the biotinylated IgE and associated complexes, leaving the unlabeled IgE in the flow-through. A reciprocal experiment was also performed in which the IgE clone being biotinylated was switched. Unsensitized BMMCs were included in both experiments as negative controls. Western blots of the immunoprecipitates from both sets of experiments revealed β-actin in the pulldown of biotinylated IgE desensitized with its corresponding Ag, but not when heterologous Ag was used for desensitization (Figure 7D). Notably, the amount of β-actin associated with desensitized IgE/FcεRI was markedly higher than that associated with Ag-challenged samples at that time point (~2 hours). Taken together, these observations suggest that Ag specificity may be achieved, at least in part, by specific association of the actin cytoskeleton with desensitized receptors. This finding also suggests that the F-actin aggregates in desensitized MCs (Figure 6A) may be at least partly associated with IgE/FcεRI.

### Manipulation of the actin cytoskeleton of MCs reverses desensitization in vitro and in vivo.

To further demonstrate the contribution of the actin cytoskeleton to MC desensitization, we attempted to reverse actin rearrangement in desensitized MCs back to their steady-state arrangement, and then examined whether this activity speedily restored their ability to mediate Ca^2+^ flux and to degranulate following exposure to Ag. To this end, we used a cell-permeable peptide construct of the *Salmonella* effector SptP, SptP^C481S^-TAT (40). SptP is secreted by *Salmonella* during infection, and has 2 effector domains: a phosphatase domain and a GAP domain. Notably, the GAP domain is necessary and sufficient to reverse the actin cytoskeletal changes caused by bacterial entry, which include ruffling and membrane protrusions, by inhibition of the small GTPases CDC42 and RAC1 (41). We have previously shown that recombinant SptP^C481S^-TAT with the catalytically inactive phosphatase domain, but a functional GAP domain, did not affect IgE-mediated MC degranulation (40). Additionally, since we observed that RAC1 is activated in desensitized MCs compared with IgE-sensitized cells (Supplemental Figure 6), we examined the effect of SptP^C481S^-TAT on this GTP-binding protein. We found that this agent reduced active RAC1 in both desensitized and unstimulated IgE-sensitized MCs to undetectable levels. Thus, SptP^C481S^-TAT appears to be acting on MCs via a RAC1-dependent pathway.

**Figure 8:**
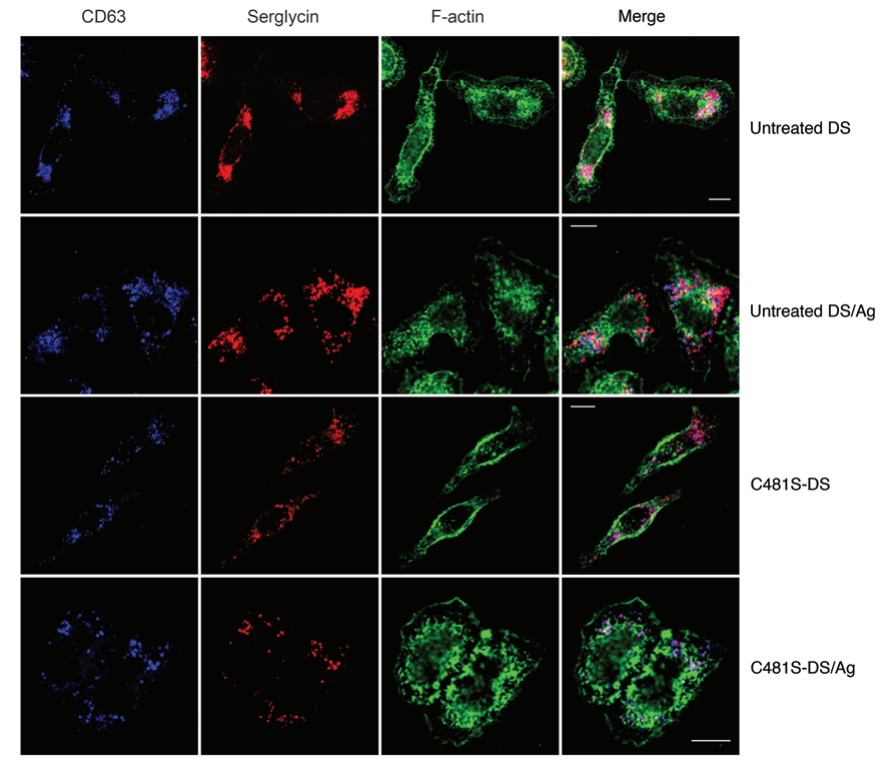
Manipulation of the actin cytoskeleton reverses actin changes caused by desensitization. IgE-sensitized serglycin-mCherry (red) RBL-2H3 cells were plated onto covered coverslips and desensitized, then treated with control or 20 μg/ml SptP^C481S^-TAT for 45 minutes before challenge with Ag for 5 minutes followed by fixation and staining for CD63 (blue) and F-actin (green). Images are representative of 3 independent experiments. Scale bars: 10 μm.

To observe cytoskeletal changes in MCs exposed to SptP^C481S^-TAT, IgE-sensitized serglycin-mCherry RBL-2H3 cells on coverslips were desensitized or control treated, then treated with SptP^C481S^-TAT or control. Thirty minutes later, cells were challenged with Ag, then fixed for immunofluorescence staining with anti-CD63 antibody and fluorescent phalloidin. Treatment with SptP^C481S^-TAT restored desensitized cells to the peripheral distribution in control RBL-2H3 cells, reducing the spreading and membrane ruffling seen in desensitized cells (Figure 8 and Supplemental Figure 7). SptP^C481S^-TAT treatment also enabled MCs to degranulate, as seen by the exocytosis of serglycin-containing secretory granules in RBL cells (Figure 8). Thus, similarly to how bacterial SptP “resets” the perturbed actin cytoskeleton in *Salmonella*-infected cells, SptP^C481S^-TAT restores actin to a basal-like distribution in MCs. This allows for treated cells to respond to Ag, both in Ag-mediated actin reorganization and also seemingly to discharge granules.

**Figure 9:**
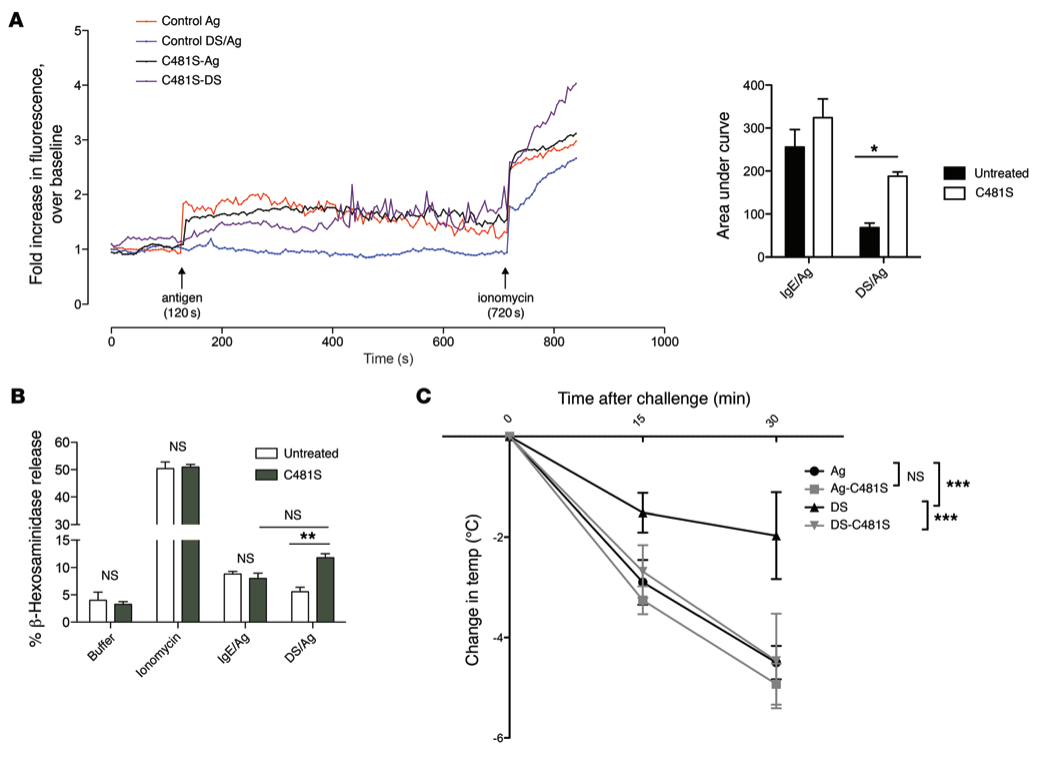
Manipulation of the actin cytoskeleton reverses desensitization in vitro and in vivo. (**A**) Control (C481S-Ag) or desensitized (C481S-DS) BMMCs were treated with SptP^C481S^-TAT (C481S-Ag, C481S-DS) or buffer (control Ag, control DS/Ag) for 30 minutes before being loaded with Fluo-4. After measurement of baseline fluorescence for 2 minutes, cells were activated with 10 ng/ml Ag and ionomycin. A representative graph is shown. AUC was averaged from 4–5 independent experiments. (**B**) Control (IgE/Ag, buffer, and ionomycin) or desensitized (DS/Ag) BMMCs were treated with 10 μg SptP^C481S^-TAT (C481S) or untreated before challenge with Ag, buffer, or ionomycin for β-hexosaminidase assay. Data are representative of at least 3 independent experiments. (**C**) IgE-sensitized mice were orally desensitized (DS, DS-C481S) or given PBS (Ag, Ag-C481S). Mice were then injected with 50 μg SptP^C481S^-TAT (Ag-C481S, DS-C481S) or equal volume PBS (Ag, DS). One hour later, mice were challenged i.p. with 500 μg Ag. Rectal temperatures were measured every 15 minutes for 30 minutes. Results were pooled from 2 independent experiments (*n* = 10). Data were analyzed via 2-way ANOVA (**A** and **B**) and repeated-measures ANOVA (**C**). Data represent SEM. *P < 0.05, **P < 0.01, ***P < 0.001.

Based on our findings, we predicted that restoring actin to the basal state using SptP^C481S^-TAT would also now allow sustained Ca^2+^ influx in desensitized cells by rescuing actin modulation of the Ca^2+^ response. To test this, BMMCs were desensitized or untreated, then incubated with SptP^C481S^-TAT or PBS before labeling with Fluo-4 for Ca^2+^ imaging. In control cells, untreated and SptP^C481S^-TAT–treated BMMCs had comparable levels of Ca^2+^ flux, while desensitized cells had no response to Ag challenge. Ca^2+^ flux in response to Ag was partially rescued in desensitized, SptP^C481S^-TAT–treated cells (Figure 9A), with a slight delay. This indicated that actin reorganization via inhibition of RAC1/CDC42 by SptP^C481S^-TAT could partially rescue the inhibition of Ca^2+^ flux caused by desensitization.

Next we sought to quantitate the effects of SptP^C481S^-TAT treatment on degranulation response of MCs. Control or desensitized cells were incubated with 10 μg/ml SptP^C481S^-TAT or PBS for 30 minutes and then challenged with Ag. As previously reported (40), SptP^C481S^-TAT treatment did not affect IgE/Ag stimulation or maximal degranulation elicited by ionomycin. Interestingly, incubation of desensitized cells with SptP^C481S^-TAT prior to Ag challenge restored their ability to degranulate in response to Ag (Figure 9B), confirming that SptP^C481S^-TAT treatment was sufficient to reverse the inhibitory effect of desensitization on Ag-mediated degranulation.

Finally, we sought to confirm our in vitro findings in our in vivo model of rapid desensitization. IgE-sensitized mice were orally desensitized as in Figure 1, or gavaged with PBS control, before being injected i.p. with 50 μg SptP^C481S^-TAT or an equivalent volume of PBS. One hour later, mice were challenged i.p. with 500 μg Ag. IgE-sensitized mice showed a dramatic (~5°C) decrease in core body temperature 30 minutes after challenge with Ag that was unaffected by SptP^C481S^-TAT treatment (Figure 9C). In contrast, desensitized mice were significantly protected from anaphylaxis, showing a less than 2°C drop in core body temperature by 30 minutes after challenge. Injection of SptP^C481S^-TAT into desensitized mice abolished this protection and rescued the anaphylactic phenotype. Cumulatively, our in vitro and in vivo data suggest that reversal of the actin cytoskeleton remodeling induced by the DS protocol on MCs is sufficient to restore their sensitivity to Ag.

## Discussion

Our studies provide new insights into the underlying mechanism of DS in MCs, which could be of immediate benefit in the design of novel strategies to induce or prolong the desensitized state of MCs for therapeutic purposes. Earlier reports have suggested that DS is the result of IgE internalization by MCs and basophils, thus preventing further responses to Ag (25, 42). These studies supposed that during DS, application of small, subactivating doses of Ag to MCs induced internalization of IgE/FcεRI complexes without triggering any degranulation events. For example, Shalit and Levi-Schaffer (42) linked refractoriness of MCs to Ag with desensitization, and hypothesized that progressive internalization of IgE with each Ag dose likely reduced the ability of the MCs to respond to the next. A more recent study by Oka et al. (25) similarly concluded that DS must occur through IgE internalization, as inhibiting IgE/FcεRI internalization by cold incubation during DS prevented effective desensitization, since these MCs degranulated when brought back to 37°C. On the other hand, Sancho-Serra et al. (26) observed that desensitized MCs had inhibited early and late MC activation responses, as well as impaired internalization of IgE/FcεRI complexes, suggesting a different explanation for MC hyporesponsiveness.

In the present study, at least 2 distinct observations suggest that depletion of IgE from the MC surface is an inadequate explanation for the hyporesponsiveness of desensitized MCs to activating doses of Ag. Firstly, we observed that desensitized MCs, both ex vivo and in vitro, are able to bind Ag, suggesting that sufficient IgE remained on cell surfaces to bind Ag after the DS protocol (Figure 2, A and B). This observation is consistent with earlier reports suggesting that ligation of as little as 10% of surface FcεRI is sufficient for a maximal MC degranulation response to Ag (43). Our own studies titrating BMMCs with various dilutions of IgE showed that maximal degranulation in response to Ag could be induced even with relatively low amounts of IgE bound to receptors (Supplemental Figure 1). Additionally, Khodoun et al. achieved desensitization of MCs using antibodies against FcεRI, without causing complete internalization of surface receptors, once again suggesting that complete depletion of surface FcεRI was not necessary for desensitization (44). Indeed, while we observed IgE internalization during Ag activation and desensitization, this typically was not complete, as Ag-activated cells still had approximately 50% of baseline cell surface IgE, whereas desensitized cells had approximately 70% of baseline cell surface IgE (Figure 2D). Secondly, we observed in vitro that desensitized MCs eventually recovered their ability to degranulate in response to Ag even without additional exposure to IgE (Figure 2C). Our finding that IgE/FcεRI complexes on desensitized MCs upon exposure to activating doses of Ag were still capable of phosphorylating several key signaling substrates also contrasts with the earlier supposition that desensitized MCs were refractory to signal transduction. Thus, our findings do not concur with any of the earlier conclusions regarding the basis for MC desensitization following DS. Instead, our studies suggest that MC hyporesponsiveness is attributable, at least in part, to abrogation of Ca^2+^ mobilization, a critical determinant of both degranulation and cytokine production responses in MCs (32).

It is noteworthy that the behavior of desensitized MCs is analogous to that of a mutant RBL-2H3 cell line, which was found to be defective in its ability to mobilize Ca^2+^ but retained normal tyrosine phosphorylation of key signaling substrates following engagement of FcεRI. In spite of their capability to transduce signals, these mutant cells, like desensitized MCs, were incapable of a degranulation response (45). Additional data linking hyporesponsiveness of MCs to their inability to mount a Ca^2+^ response come from the observation that desensitized MCs can be induced to degranulate by artificial induction of Ca^2+^ influx using ionomycin (Figure 4B).

We have deduced that the reason for the lack of a Ca^2+^ response in desensitized MCs in spite of their capability to mediate FcεRI-mediated signal transduction is the marked alteration of the cellular distribution of the actin cytoskeleton. In our hands, serial exposure of MCs to increasing amounts of subactivating doses of Ag during DS resulted in the significant redistribution of F-actin. Remarkably, the remodeled actin cytoskeleton persisted even after DS and when the desensitized cells were challenged with a further activating dose of Ag, suggesting these actin complexes were highly stable.

There is now a growing realization that actin, specifically its organization and spatial distribution, plays a critical role in regulating Ca^2+^ mobilization in a variety of cell types, including T cells (20), platelets (46), and, importantly, MCs (47). Although the precise mechanism of how the actin cytoskeleton modulates Ca^2+^ flux in MCs remains to be elucidated, studies in other cell types suggest that actin modulates the association of the endoplasmic reticulum Ca^2+^ sensor stromal interaction molecule 1 (STIM1) with the plasma membrane Ca^2+^ channels ORAI1 and TRPC (48). This association typically precedes the formation of a functional Ca^2+^ release–activated channel (CRAC) on the plasma membrane that initiates the influx of extracellular Ca^2+^. Support for this proposed role of actin rearrangement comes from our observation that upon reversing actin cytoskeletal organization in desensitized MCs with a cell-permeable construct of the *Salmonella* effector SptP, SptP^C481S^-TAT, we could restore its Ca^2+^ mobilization and degranulation activity as well as the anaphylactic phenotype in desensitized mice. This is a highly accelerated recovery of degranulation in desensitized MCs, which without further manipulation took 48 hours to regain responsiveness to Ag challenge (Figure 2C). In these recovered desensitized cells, we have also observed reversion of F-actin organization to the naive IgE-sensitized phenotype (data not shown), further suggesting that proper localization of actin is important for IgE/Ag activation. Presumably, the effect of SptP^C481S^-TAT on desensitized MCs occurs via the known GAP activity of SptP^C481S^-TAT on RAC1/CDC42, as we have observed that this pathway is activated during desensitization and is inhibited by SptP^C481S^-TAT (Supplemental Figure 6). Curiously, RAC1/CDC42 has been postulated to have a positive rather than a negative regulatory effect on Ca^2+^ mobilization in MCs as would be expected from our model (49). Conceivably, RAC1/CDC42 could be exerting its effect in our system via other pleiotropic downstream effects on actin cytoskeletal rearrangement (Figure 8), possibly via cofilin inactivation (phosphorylation) downstream of PAK kinases (50), rather than through direct effects on Ca^2+^ mobilization via IP_3_ production (49).

Our observed absence of sustained Ca^2+^ mobilization during the desensitization process (Figure 5B) also explains the lack of degranulation during DS, despite the incomplete displacement of the actin cytoskeleton prior to completion of desensitization. We postulate that the subactivating doses of Ag applied during DS did not result in a sufficient magnitude of signal transduction to provoke a sustained Ca^2+^ flux, while allowing for progressive actin cytoskeletal rearrangements such that the fully desensitized MC was no longer able to respond appropriately to activating doses of Ag.

We have also been able to confirm the Ag specificity of the desensitization protocol (Figure 7A). While a model of desensitization that involves the actin cytoskeleton may appear nonspecific with regard to which receptors are impacted, there is specificity in the way desensitized receptors interact with the actin cytoskeleton, which could, at least in part, explain the Ag specificity of DS. We found that desensitization to 1 particular Ag resulted in the specific association of actin with homologous, and not heterologous, desensitized receptors (Figure 7D). Since the actin cytoskeleton can regulate Ca^2+^ responses, the stable aggregates of actin filaments in desensitized MCs could negatively regulate the ability of the desensitized receptor to mediate a Ca^2+^ response. Although an association between actin and FcεRI was observed during Ag-induced MC degranulation, this interaction was transient (Figure 7, B and C), and, importantly, it seemed to peak at least 30 minutes after activation, which suggests that rather than promoting signal transduction, this association between FcεRI and actin may serve to terminate specific signaling processes.

With the ever-increasing incidence of allergic reactions in the population to constituents in food and essential drugs and the lack of alternatives to DS protocols, there is a dire need to understand correctly the underlying basis for MC desensitization. Our studies have uncovered, at least in part, the basis for DS-induced MC desensitization. While we cannot rule out the possibility of other contributing factors such as negative regulatory signaling, our observations strongly indicate a role for aberrant actin remodeling during desensitization and the resulting inhibition of Ca^2+^ mobilization in the inhibition of MC degranulation in response to subsequent Ag challenge. We also present a model of passive systemic anaphylaxis and oral desensitization in vivo. While our model cannot be considered wholly mast cell specific due to the caveats of the Wsh mouse (51), our in vitro and in vivo data indicate that this cell type is likely to be the main effector in desensitization. Additionally, our oral in vivo desensitization model could be of value for future studies on mechanisms of desensitization, as it is a more convenient and humane way to serially administer Ag compared with repeated-injection protocols. We believe these studies provide valuable insights for the development of new strategies to either induce MC desensitization or prolong its effects.

## Methods

### Antibodies, cell lines, and reagents.

Anti–phospho-LAT (Y226) (catalog 07-295), anti-FcεRIγ (06-727), and anti-LAT (06-807) antibodies were from Upstate Technology. Phospho-LYN (Y396) antibody was from Abcam (ab40660). Antibodies against phospho-cofilin (mAb 3313) and total cofilin (mAb 5175), as well as total LYN (catalog 2796), were from Cell Signaling Technologies. Antibodies against phospho-ERK (sc-7383) and ERK2 (sc-154) were from Santa Cruz Biotechnology. Anti–β-actin antibody was from Sigma-Aldrich (clone AC-74). BMMCs were obtained from femur bone marrow of 8-week-old C57BL/6 mice and cultured in BMMC medium (RPMI-1640 with l-glutamine, 10% heat-inactivated FBS, 10 mM HEPES, 1 mM sodium pyruvate, 1× nonessential amino acids, and 100 U/ml penicillin/streptomycin) at a density of 0.5 × 10^6^ to 1 × 10^6^ cells/ml with 5 ng/ml recombinant murine IL-3 (R&D Systems) and 5% to 10% stem cell factor-containing supernatant from CHO-KL cells for 4–6 weeks until maturation. CHO-KL cells were a gift from M. Arock (Laboratoire de Biologie et Pharmacologie Appliquée, Paris, France). RBL-2H3 cells were purchased from ATCC and maintained in MEM with Earle’s salts, l-glutamine, 1 mM sodium pyruvate, and 15% heat-inactivated FBS. TNP(12)-OVA was obtained from Biosearch Technologies. All other chemical reagents were from Sigma-Aldrich unless otherwise specified.

### In vitro desensitization and β-hexosaminidase assay.

All experiments were repeated with both anti-DNP IgE (SPE-7, Sigma-Aldrich) and anti-TNP IgE (BD Biosciences) unless otherwise stated. BMMCs or RBL-2H3 cells were sensitized with 0.25–0.5 μg/ml IgE for 4 hours to overnight and washed 3 times before the start of the experiment. All assays were done in Tyrode’s buffer (135 mM NaCl, 5.5 mM glucose, 5 mM KCl, 1 mM MgCl_2_, 1.3 mM CaCl_2_, 0.5% BSA, pH 7.4) unless otherwise specified. For β-hexosaminidase assay, IgE-sensitized BMMCs were seeded at 3 × 10^5^ to 5 × 10^5^ cells per well in V-bottomed 96-well plates on the day of the experiment. RBL-2H3 cells were seeded at 5 × 10^4^ cells per well in MEM with IgE in 96-well flat-bottomed plates overnight. Assays were then performed as previously described (40).

### Intracellular cytokine staining, ELISAs, and RT-PCR.

In each case, 5 × 10^5^ BMMCs per sample were sensitized with anti-DNP IgE and desensitized or challenged with Ag, then incubated in 3 μg/ml brefeldin A (eBioscience) for an additional 6 hours (total time ~8 hours). The cells were then washed, permeabilized, blocked with 0.25% saponin in 1% BSA in PBS, and stained for intracellular CCL2 and TNF-α using FITC anti–mouse CCL2 and allophycocyanin anti–mouse TNF-α (eBioscience) before assay via FACS. To assay cytokine secretion, untreated, desensitized, or Ag-challenged BMMCs (5 × 10^5^ BMMCs per sample in triplicate) were incubated in RPMI for 24 hours. Cell culture supernatants were diluted 1:5 and assayed for CCL2 and TNF-α using a kit (eBioscience) according to the manufacturer’s instructions. For cytokine mRNA quantitation, RNA was prepared from 5 × 10^5^ BMMCs per sample in triplicate. RNA was isolated using an RNeasy purification system (Qiagen). cDNA was synthesized using the iScript cDNA synthesis kit (Bio-Rad). SYBR Green and iCycler machine (Bio-Rad) were used for real-time PCR. Primers were obtained from IDT-DNA for β-actin (*Actb*): 5′-TGAGAGGGAAATCGTGCGTGACAT, 5′-ACCGCTCATTGACGATAGTGATGA; CCL2: 5′-AGCAGGTGTCCCAAAGAAGCTGTA, 5′-AAAGGTGCTGAAGACCTTAGGGCA; *Tnfa*: 5′-TCTCATGCACCACCATCAAGGACT-3′, 5′-ACCACTCTCCCTTTGCAGAACTCA-3′. All target gene RNA expressions were normalized to *Actb* expression. Data were expressed as fold over IgE-only controls.

### Mice and in vivo model of DS and systemic anaphylaxis.

Six- to eight-week-old female C57BL/6 mice were purchased from The Jackson Laboratory. All experiments were performed in accordance with Duke University’s Animal Care and Use Committee. Mice were sensitized i.p. with 10 μg of anti-TNP IgE (BD Pharmingen) in 100 μl sterile PBS 1–7 days before the experiment. Desensitization doses were delivered via oral gavage in a volume of 100 μl in saline and were spaced 30 minutes apart. Control animals received PBS gavage. For Ag challenge, mice were given 500 μg of TNP-OVA (Protein Sciences) i.p. Core body temperature was monitored with a rectal thermometer before each desensitization dose, and every 15 minutes after challenge with Ag for up to 2 hours. For assay of peritoneal cells via FACS or histology, mice were lavaged with 4 ml cold PBS containing 1 mM EDTA. To assay Ag binding, 3 × 10^6^ peritoneal lavage cells were blocked in 1% BSA in PBS before incubation with 100 ng/ml Alexa Fluor 647–labeled TNP-OVA prepared using a protein labeling kit (Molecular Probes) according to the manufacturer’s instructions. After incubation on ice for 1 hour with labeled Ag, cells were washed 4 times in cold PBS before fixation with 4% paraformaldehyde and assay via FACS. For visualization of MC degranulation, 100 μl of peritoneal lavage was cytospun onto charged glass slides at 600 rpm for 5 minutes. Slides were fixed in Carnoy’s fixative (60% ethanol, 30% chloroform, 10% glacial acetic acid) before staining with toluidine blue dye.

### Immunofluorescence.

RBL-2H3 cells were seeded onto clean glass coverslips in IgE-containing medium. Cells were then stained for cytoskeleton using methods previously described (52). Briefly, cells were rinsed in cytoskeletal buffer (CSK buffer) (0.1 MES, pH 6.9, 2 mM EGTA, 2 mM MgCl_2_, 4% polyethylene glycol 8000) before being fixed with 3.7% formaldehyde in CSK buffer, then extracted with 0.5% Triton-X in CSK buffer before staining with antibodies. Coverslips were mounted in ProLong Gold (Life Technologies) to preserve fluorescence before imaging using a Nikon ECLIPSE TE200 laser scanning confocal microscope, using a sequential channel series approach to avoid spectral overlapping. Images were edited using ImageJ software (NIH).

### Biotin labeling, immunoprecipitation, SDS-PAGE, and protein immunoblotting.

IgE was biotinylated using a kit (Thermo Fisher Scientific) according to the manufacturer’s instructions. For immunoprecipitation of biotinylated IgE, 10^7^ BMMCs sensitized with a mixture of biotinylated and unbiotinylated IgE per sample were lysed in immunoprecipitation lysis/wash buffer (Pierce) containing protease inhibitors. After clearing of cell debris, 80 μl of streptavidin agarose bead slurry (Thermo Fisher Scientific) was added to 500 μg of total protein and incubated at 4°C for 2 hours. The beads were then washed 5 times with lysis/wash buffer, then boiled with 50 μl 2× SDS-PAGE buffer before electrophoresis. For analysis of phosphotyrosine signaling, 2 × 10^6^ BMMCs per sample were lysed directly in 4× Laemmli sample buffer containing β-mercaptoethanol and immediately boiled for 5 minutes. Samples were loaded on 4%–20% acrylamide gels for SDS-PAGE before transfer and immunoblotting with respective antibodies to phospho-proteins. In some cases, after exposing of blots, membranes were stripped and reprobed for total protein.

### Calcium measurements.

BMMCs in RPMI were loaded with 1 μM Fluo-4 AM (Life Technologies) with 0.02% Pluronic F-127 for 10 minutes at 37°C, washed twice in Tyrode’s buffer, and recovered in Tyrode’s buffer (135 mM NaCl, 5.5 mM glucose, 5 mM KCl, 1 mM MgCl_2_, 1.3 mM CaCl_2_, 0.5% BSA, pH 7.4) for 15 minutes at 37°C before imaging. Images were acquired every 5 seconds on an Andor XD revolution spinning disk confocal microscope (Olympus), and fluorescence was quantitated using MetaMorph software (Molecular Devices). Data were expressed as fold over baseline fluorescence. Cells that moved out of focus during the experiment or did not respond to a final administration of ionomycin were excluded from analysis.

### Constructs, transfection, and protein purification.

We used a His-tagged, phosphatase-inactive, cell-permeable form of SptP (SptP^C481S^-TAT) that has been described (40). Recombinant His-tagged SptP^C481S^-TAT expressed in BL21-Gold–competent cells (Agilent Technologies) was purified using a cobalt column (His Gravitrap Talon, GE Healthcare) and extensively dialyzed in PBS to remove salts before concentrating and quantitation of protein. Serglycin-mCherry–expressing RBL-2H3 cells were created by retroviral transduction. Transduced cells were selected with 500 μg/ml geneticin.

### Statistics.

All data are expressed as SEM. Significance was calculated by unpaired 2-tailed Student’s *t* test to assess data with 2 groups or by 1- or 2-way ANOVA and Tukey’s multiple comparison or Bonferroni post-tests, as appropriate, for all other experiments. Results were considered significant if *P* was less than 0.05. All graphs and analyses were done with Prism (GraphPad) software.

### Study approval.

All studies were performed with the approval of the Duke University Animal Care and Use Program.

## Author contributions

AMC and SNA conceived of the study. WXGA and SNA planned most of the experiments, with suggestions by AWB, HWC, and MK. WXGA performed most of the experiments. AMC contributed to experiments. HWC synthesized constructs used in this study. All authors contributed to the writing of the manuscript.

## Supplementary Material

Supplemental data

## Figures and Tables

**Table 2:**
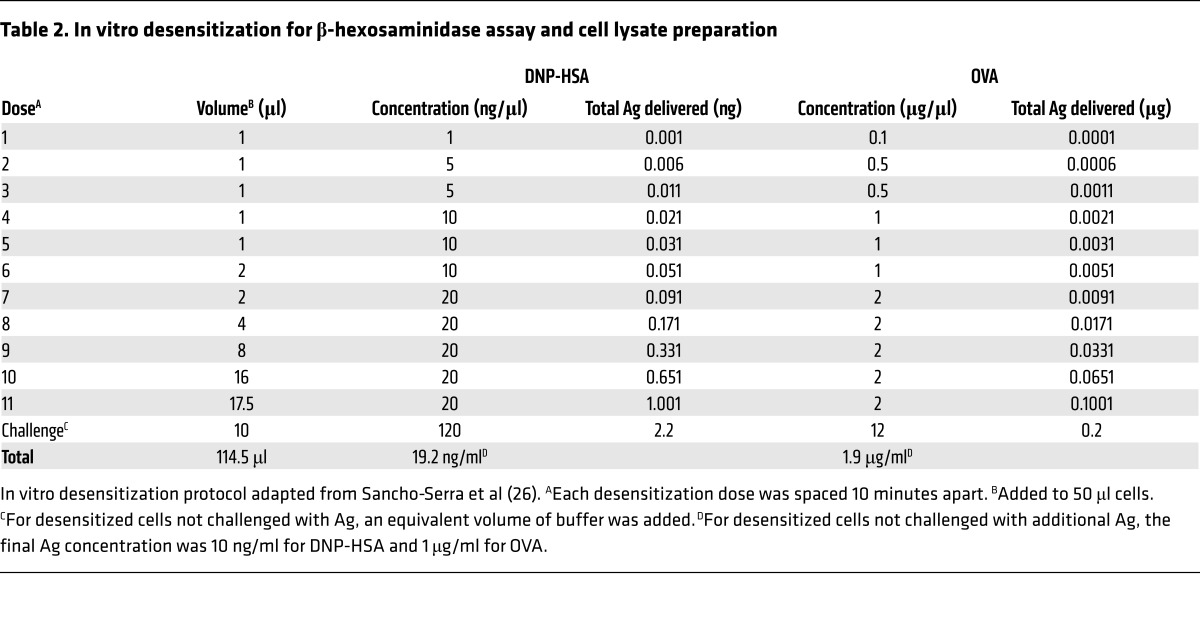
In vitro desensitization for β-hexosaminidase assay and cell lysate preparation

**Table 1:**
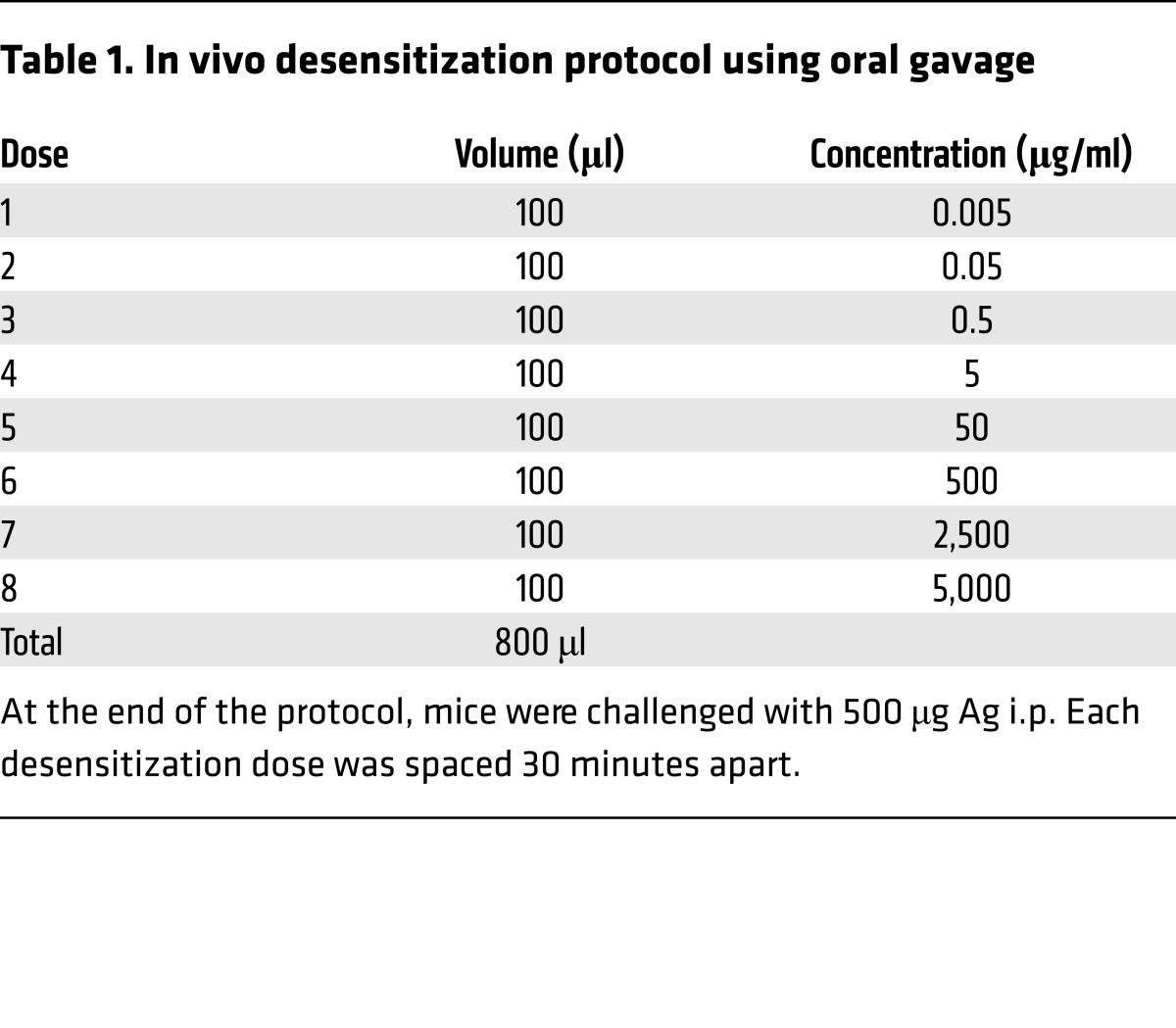
In vivo desensitization protocol using oral gavage
