# Supplementary material for: Mast cell desensitization inhibits calcium flux and aberrantly remodels actin
Source: J Clin Invest. 2016 Sep 26;126(11):4103–18. doi: 10.1172/JCI87492 (PMC5096925; doi:10.1172/JCI87492)
Supplement: Supplemental data [file jci-126-87492-s001.pdf]

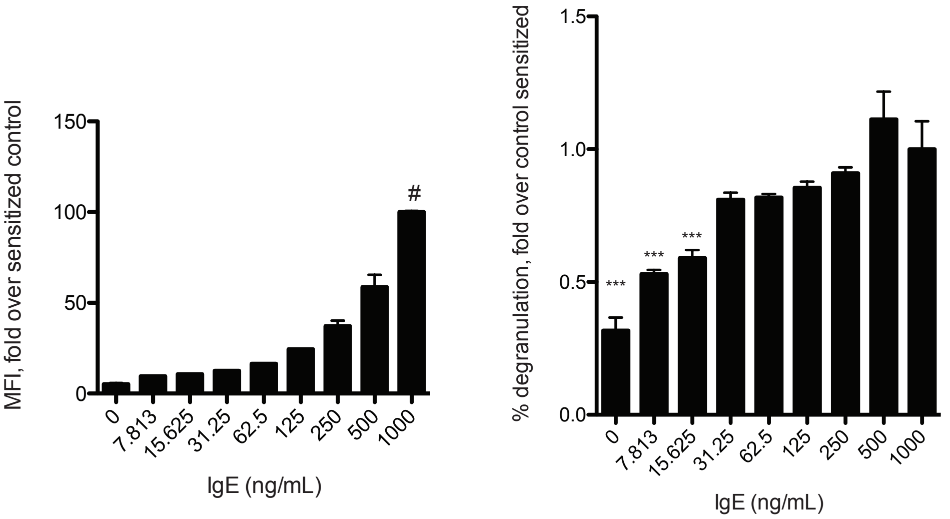

Supplementary Figure 1. Degranulation does not scale proportionately with Ag binding. $5X10^5$ BMMCs per well in quadruplicate were sensitized wtih various concentrations of anti-TNP IgE, then assayed for A647-labelled TNP-OVA binding via FACS (left) or challenged with 10ng/mL TNP-OVA to assay degranulation (right). Data were expressed as fold over positive control (1000ng/mL IgE). Data are representative of 2 independent experiments. Error bars indicate SE of mean. ***: $p<0.001$ compared to 1000ng/mL group. #: $p<0.001$ compared to all other groups as determined by one-way ANOVA.

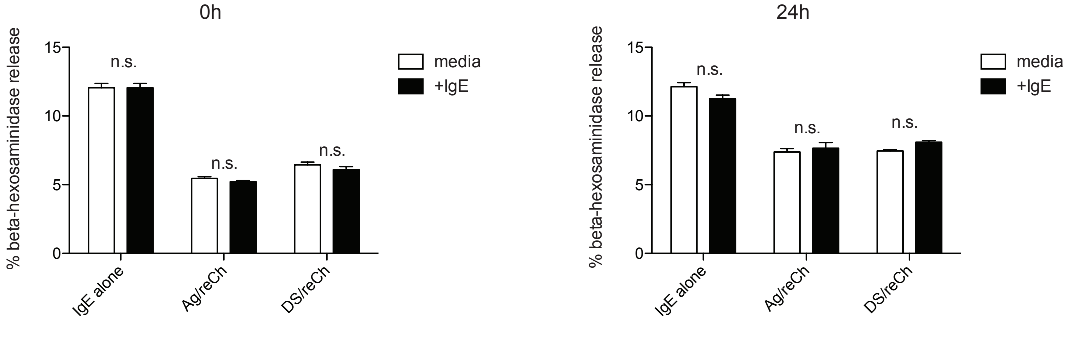

Supplementary Figure 2. Re-sensitization with IgE does not restore the ability of desensitized BMMCs to degranulate. Untreated (IgE/Ag), Ag-treated (Ag/reCh) or desensitized (DS/reCh) BMMcs were incubated for 0 or 24h, washed, then incubated for 2h with or without IgE (0.5μg/mL) before challenge with Ag. Degranulation was measured using β-hexosaminidase assay. Data represent SE of mean. n.s.: not significant between media and +IgE groups, as determined via ANOVA.

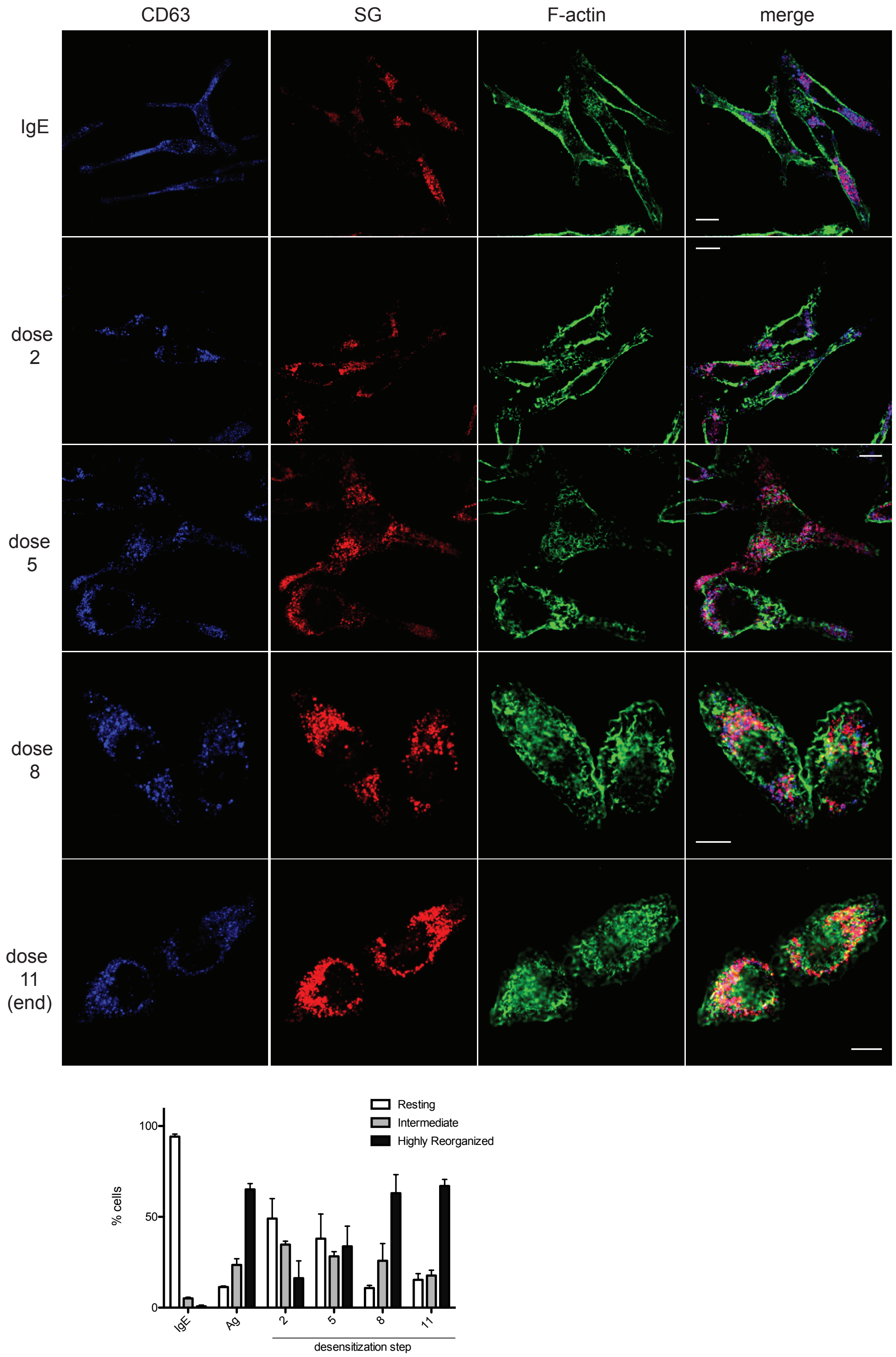

Supplementary Figure 3. Actin reorganization during desensitization. RBL-2H3 cells expressing mCherry-serglycin (SG) (red) were seeded onto coverslips. At various stages of desensitization (out of 11), cells were fixed and stained for CD63 (blue) and F-actin (green). Images were taken at 100x magnification and are representative of 3 independent experiments. Scale bar (grey) indicates 10μm. The various F-actin phenotypes of cells in 5 random fields were counted at 60x and expressed as % of total cells and averaged between 3 experiments. Error bars indicate SE of mean.

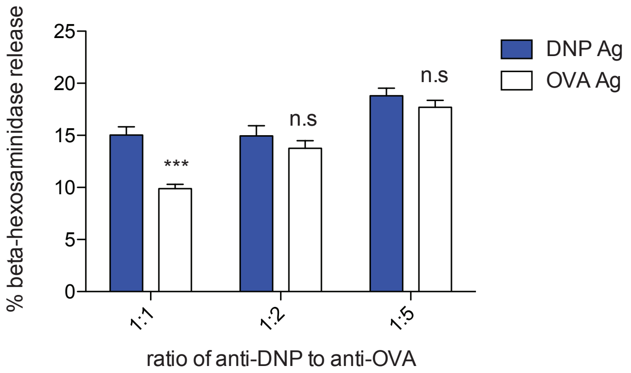

Supplemental Figure 4. Determining optimal ratios of IgE for MC activation. RBL-2H3 cells were sensitized with mixtures of anti-DNP IgE and anti-OVA IgE of varying ratios, then challenged with 10ng/mL DNP-HSA (DNP Ag) or 1µg/mL OVA (OVA Ag). Degranulation was measured via beta-hexosaminidase assay. Data indicate SE of mean. ***: $p<0.005$, n.s: not significant.

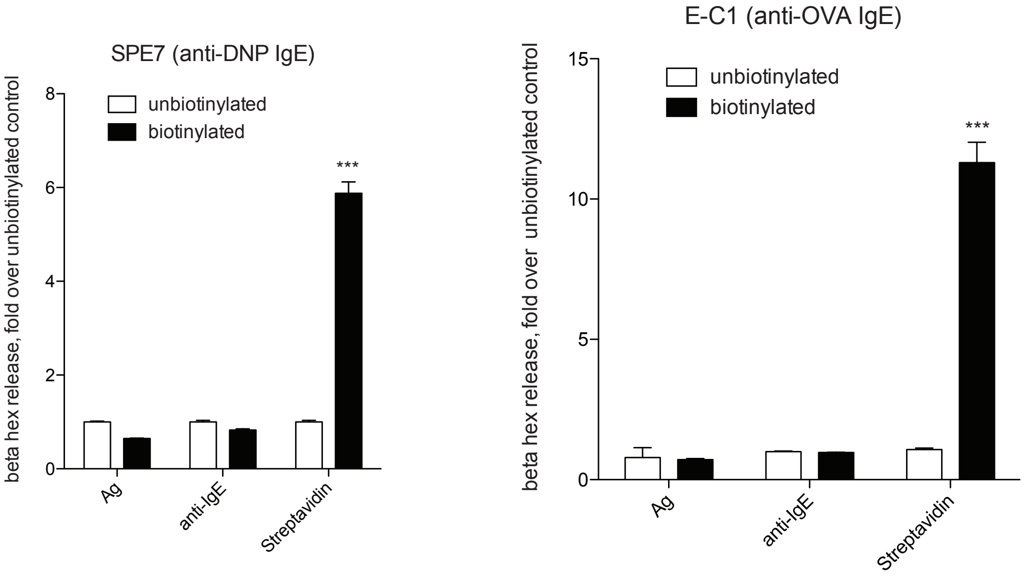

Supplementary Figure 5. Biotinylated IgE is functional. Biotinylated or unlabeled anti-DNP IgE (SPE7 clone) or anti-OVA IgE (E-C1 clone) were incubated with BMMCs overnight. After washing with Tyrode's buffer, sensitized cells were challenged with respective antigen (Ag) at 10ng/mL for anti-DNP IgE or 5μg/mL for anti-OVA IgE, 1μg/mL anti-IgE or 0.5 μg/mL streptavidin. Data represent SE of mean. ***:$p<0.005$ as determined by ANOVA, with comparisons between unbiotinylated and biotinylated groups.

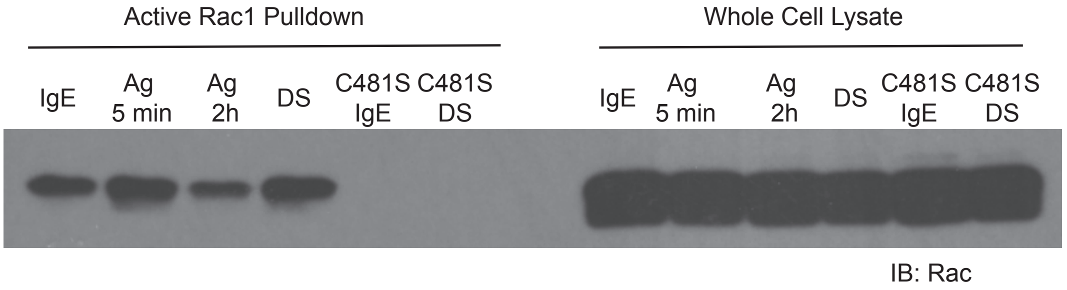

Supplementary Figure 6. Rac activation during desensitization. Lysates were prepared from untreated, Ag-challenged or desensitized BMMCs treated or untreated with SpTP^{C481S}-TAT, then immunoprecipitated for active Rac1 using GST Pak1-PBD bound to glutathione agarose beads. Immunoprecipitates and control whole cell lysates were then immunoblotted with anti-Rac antibody.

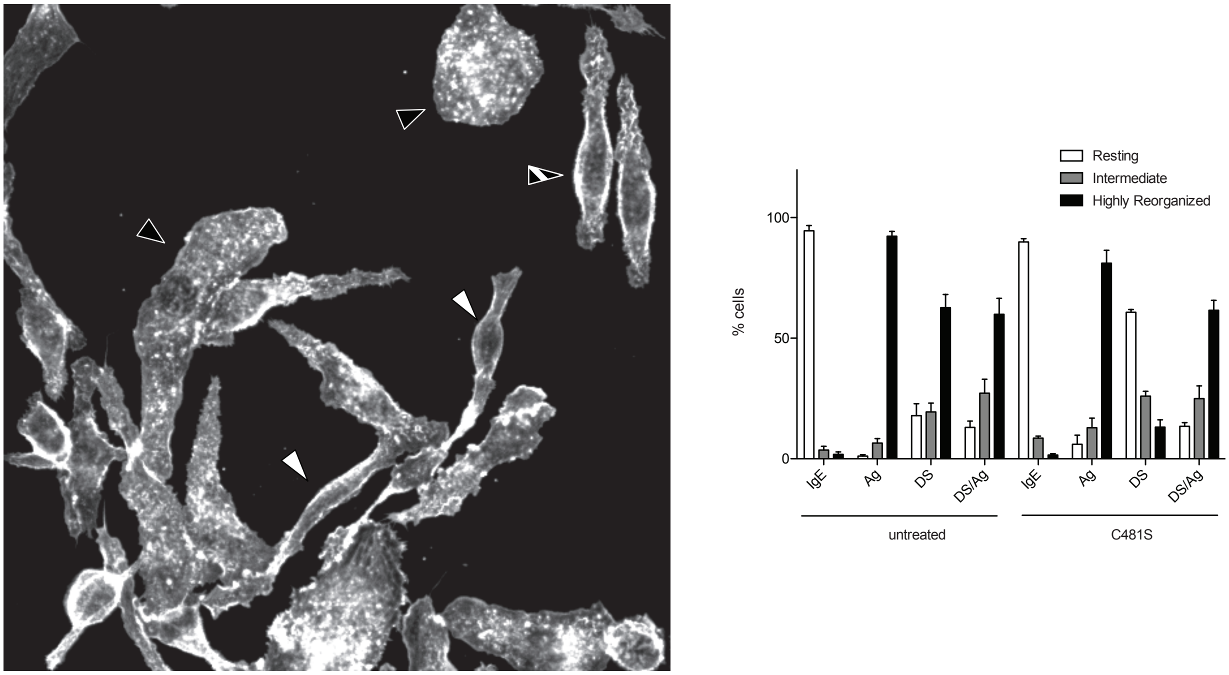

Supplemental Figure 7. Percentage of actin reorganized cells with desensitization and SptP^{C481S}-TAT treatment. RBL-2H3 cells were seeded onto coverslips and left untreated (IgE), challenged with Ag (Ag) desensitized (DS) or desensitized and subsequently Ag-challenged (DS/Ag). For SptP^{C481S}-TAT -treated cells (C481S), cells were incubated with 10µg SptP^{C481S}-TAT before Ag challenge. Cells were fixed and stained with A647-phalloidin and imaged at 60x. The number of cells showing resting-type, intermediate or highly reorganized actin phenotypes were counted in 5 random fields per sample. Counts were averaged between 3 independent experiments. An example image showing the various phenotypes is shown. White arrows: resting, hatched arrows: intermediate, empty arrows: highly reorganized. Data represent SE of mean.
